# From sports UGC to wanting to watch live: does seeing others in user-generated photos change spectating intentions?

**DOI:** 10.3389/fpsyg.2026.1788581

**Published:** 2026-05-29

**Authors:** Banghan Xu, Jialiang Chen

**Affiliations:** 1 School of Economics and Management, Shanghai University of Sport, Shanghai, China; 2 School of Psychology, Shanghai University of Sport, Shanghai, China

**Keywords:** mental simulation, photo presentation, social media, spectating intention, user-generated content

## Abstract

**Introduction:**

Social media has become a core arena for contemporary sports marketing, where sports-related user-generated content increasingly shapes how fans perceive events and form engagement intentions. Among sports UGC, stadium photos shared by ordinary users are especially prevalent. However, limited research has examined how visual cues within these images influence spectators’ intentions to attend or watch live sports events. Addressing this gap, the present research investigates whether human presence in sports-related user-generated photos changes spectators’ intentions and through which psychological mechanism this effect occurs.

**Methods:**

Drawing on self-referencing and mental simulation theories, this research proposes that human presence in sports UGC facilitates viewers’ imagination of being at the event, thereby enhancing spectating intentions. Across three experiments using authentic sports-related user-generated images, the study examined the direct effect of human presence on spectating intention, the mediating role of mental simulation, and the moderating role of stadium context.

**Results:**

Study 1 showed that sports UGC photos containing people generated significantly stronger spectating intentions than photos without people. Study 2 demonstrated that this effect was mediated by mental simulation, indicating that human presence increased spectating intentions by enabling viewers to mentally simulate the live sports experience. Study 3 further revealed the moderating role of stadium context. The indirect effect of human presence through mental simulation was stronger in fan-oriented contexts that emphasized social interaction and shared emotions than in competition-oriented contexts that focused on athletic performance.

**Discussion:**

By tracing a psychological pathway from scrolling through sports UGC to wanting to watch live sports, this research advances understanding of visual engagement and fan behavior in the evolving sports marketing landscape. The findings offer actionable insights for sports marketers, event organizers, and digital platforms seeking to leverage user-generated imagery to enhance fan engagement and drive live sports consumption.

## 1 Introduction

Against the backdrop of rapid developments in digital media, social media has become a central platform through which users access sports-related information, construct expectations about spectating, and form behavioral intentions (Morgan et al., 2021; Tsai et al., 2021). Particularly on image-oriented social media platforms such as Weibo, Xiaohongshu, and Instagram, the volume and circulation of user-generated content have expanded substantially. Within this context, stadium photos stand out as an important visual resource shaping spectators’ intentions, owing to their immersive situational qualities and capacity for emotional expression (Symons et al., 2024). Existing research has begun to examine the effects of user-generated images in domains such as tourism (Aboalganam et al., 2025), food service (Hassan and Chee, 2024), and retailing (Li et al., 2021). However, within the context of sports spectating, how people depicted in stadium photos are perceived by users and how such perceptions translate into spectating behavior remain insufficiently examined. This gap constrains a deeper understanding of the role that visual sports UGC plays in users’ behavioral decision-making.

From a cognitive psychological perspective, whether information elicits deep processing largely depends on the extent to which it is connected to an individual’s self-concept. The self-referencing effect suggests that when external information is linked to one’s own experiences, its psychological significance is enhanced, thereby fostering deeper cognitive processing and more enduring behavioral tendencies (de Graaf, 2023; Gong et al., 2025). In visual communication contexts, people depicted in images function as intentional and socially meaningful cues that increase the self-relevance of visual content, thereby triggering self-referential processing (Yim et al., 2021). Prior research has shown that the presence of people in images not only enhances perceived realism but also facilitates role simulation and emotional immersion in depicted scenarios, which may subsequently influence behavioral judgments (Huang et al., 2023; Jin and Youn, 2023).

Building on this perspective, mental simulation theory posits that individuals construct anticipatory representations of future situations based on prior experiences when encountering external stimuli. Such simulation is not merely an imaginative process but a critical cognitive mechanism through which individuals preconfigure behavioral decision pathways (Kappes and Morewedge, 2016). In studies of tourism imagery, the presence of people has been shown to strengthen viewers’ imagination of future travel experiences, thereby increasing destination attractiveness and visitation intentions (Zhou et al., 2024). Despite these insights, no research has systematically examined whether people depicted in sports spectating–related UGC photos similarly influence users’ spectating intentions through mental simulation. This absence limits current understanding of the deeper cognitive pathways underlying users’ processing of sports-related images.

Moreover, sports spectating is characterized by both task-oriented elements and strong social interaction features, suggesting that people depicted in different stadium contexts may elicit distinct psychological processing modes. Images centered on competition scenes tend to convey action structures and goal-oriented dynamics, whereas images emphasizing fan interaction foreground emotional expression and collective belonging. Such contextual differences may activate different self-projection routes and simulation processes, thereby influencing both the strength and direction of how human cues affect spectating intentions (Li et al., 2024). Accordingly, it is necessary to incorporate stadium context as a moderating factor to reveal how visual context conditions the psychological effects of human presence. This perspective responds to recent theoretical calls in UGC visual research to account for the interactive effects of complex image cues.

This research offers several theoretical contributions. First, by integrating the self-referencing effect with mental simulation theory, it advances understanding of the cognitive processing pathways through which visual content induces behavioral intentions in social media environments. Second, by extending psychological mechanisms primarily examined in advertising and tourism contexts to the domain of sports visual UGC, this study broadens the applicability of information-processing theories within social media communication and addresses an important gap in sports communication research. From a practical standpoint, the findings provide empirical guidance for event organizers, platform operators, and sports marketing practitioners regarding image content optimization and recommendation system design. By identifying which combinations of human presence and stadium context are more effective in enhancing spectating intentions, the results offer content-level intervention insights for image selection, presentation, and algorithmic dissemination, thereby improving the behavioral conversion efficiency of sports UGC.

The novelty of this research is reflected in three key aspects. First, it employs authentic stadium images uploaded by users as research stimuli, closely aligning with the natural content ecology of social media platforms and enabling examination of users’ cognitive and behavioral responses in ecologically valid settings. Second, the study jointly considers self-referencing and mental simulation as coexisting psychological processes within a unified theoretical framework, emphasizing the cognitive mechanisms that operate between information exposure and behavioral response. Third, by introducing stadium context as a critical factor shaping audience responses, the research reveals the heterogeneity and complexity underlying the relationship between visual content and spectating intentions, offering a valuable reference for future investigations of visual communication effects from multidimensional perspectives.

## 2 Theoretical background and hypothesis development

### 2.1 Self-referencing effect

The self-referencing effect refers to individuals’ tendency to relate external information to their own cognitive structures, emotional experiences, or life events during information processing, thereby deepening comprehension, enhancing psychological significance, and improving memory retention (Mattavelli et al., 2017). When information content is highly relevant to an individual’s self-concept or lived experience, it is more likely to elicit emotional resonance and to be processed at a higher cognitive level. This mechanism has been widely applied in advertising and social media communication, where prior research demonstrates that self-referencing cues not only enhance attention to information but also strengthen users’ emotional responses, ultimately facilitating the formation of behavioral tendencies (Phua and Kim, 2018; Ku et al., 2019).

Within the advertising literature, the self-referencing effect has been identified as a key psychological mechanism underlying user engagement, attitude change, and the formation of behavioral intentions. A growing body of empirical evidence indicates that advertising messages containing self-relevant cues can significantly enhance positive emotional reactions and participation intentions. For example, food-related video blogs on social media that successfully evoke self-connections have been shown to increase viewers’ emotional responses and advertising acceptance (Elshazly et al., 2025). Other studies suggest that information presentation strategies integrating self-referential structures with situational framing not only improve persuasive effectiveness but also promote active cognitive processing and content retransmission intentions (Zhang et al., 2020). In addition, humorous narrative advertising has been found to enhance favorable attitudes toward advertisements by increasing entertainment value, self-connection, and perceived interactivity, with advertising engagement playing a critical moderating role in this process (Untarini et al., 2022).

In recent years, as visual communication and tourism advertising have continued to expand, the self-referencing effect has been increasingly applied in destination marketing and image-driven communication contexts. Research indicates that when tourism advertising content aligns with users’ self-concepts, it can elicit stronger emotional responses and significantly enhance interest in and intentions to visit destinations (Wang et al., 2024). For instance, in the promotion of cultural tourism destinations, tourists are more likely to establish self-connections with virtual endorsers, whereas in nature-based tourism contexts, human endorsers are more effective in eliciting emotional identification. Regardless of endorser type, their influence on visitation intention operates through a serial mediation mechanism involving credibility and self-referencing (Meng et al., 2025). Other studies further demonstrate that when image-based user-generated advertisements are congruent with users’ visual styles and self-awareness structures, audience attitudes and interests increase significantly (Wang et al., 2024).

Beyond its extensive application in advertising and tourism research, the role of the self-referencing effect in social media–based visual user-generated content has received growing scholarly attention. Prior studies suggest that human cues, as the most socially salient and intentional elements within images, can activate users’ imagination of similar personal experiences, thereby strengthening the perceived relevance between information content and the self-concept (Huang et al., 2023). This form of self-related processing not only enhances the psychological accessibility of UGC content but also increases users’ sense of immersion and identification with the depicted scenario, which may subsequently influence expectations about future behavior and participation intentions.

In particular, recent research emphasizes that in sports-related social media contexts, the manner in which people are depicted in images may serve as a critical trigger for users to simulate their own spectating experiences. By evoking self-related memories and imaginative projections, human presence in sports UGC can guide users in constructing subjectively realistic behavioral scenarios, thereby indirectly shaping their spectating intentions (Li and Wan, 2025). These findings suggest that self-referencing provides an essential cognitive foundation for understanding how visual human cues embedded in sports UGC influence users’ behavioral decision-making.

### 2.2 Mental simulation theory

Mental simulation refers to individuals’ internal representation and rehearsal of possible future situations, behavioral processes, or outcomes when exposed to external informational stimuli. This psychological representational activity involves the perception, reconstruction, and projection of multidimensional information, including visual, emotional, and action-related cues, and constitutes a core cognitive mechanism linking perceptual inputs to behavioral responses (Petit et al., 2022). Within the fields of information processing and consumer behavior, mental simulation is regarded as a higher-order cognitive process that enhances both the depth and precision of information processing while increasing individuals’ sense of immersion and self-involvement in visual or verbal content (Park and Yoo, 2020; Yim et al., 2021).

In social media contexts, user-generated content is predominantly presented in the form of images, short videos, and text, among which images serve as a particularly powerful trigger for mental simulation due to their concreteness and immediacy. Compared with idealized narrative scenarios constructed in commercial advertising, UGC images are more closely aligned with everyday life and realistic contexts, making them more likely to elicit viewers’ psychological projection and behavioral imagination of personally experiencing the depicted situation (Zhao et al., 2011; Correia et al., 2025). The concreteness and structural completeness of situational cues largely determine whether individuals engage in simulation construction, which in turn is closely associated with attitude evaluation, emotional arousal, and the formation of behavioral tendencies (Kim et al., 2014; Gunden et al., 2021).

At a more granular level, the mechanisms through which UGC images evoke mental simulation can be examined across multiple dimensions. First, key visual elements within images, such as people, actions, and environmental backgrounds, provide the foundational framework for simulation construction (Zheng et al., 2024). Image content characterized by interaction or emotional expression facilitates subjective immersion and scenario reenactment, thereby activating simulation-oriented thoughts such as “what it would feel like if I were there” or “how I would respond in that situation” (Ying et al., 2022). Such reenactment processes not only intensify cognitive engagement but also strengthen emotional connections and motivational activation (Poirier et al., 2024). Second, image composition and perspective features play a critical role in regulating the intensity of mental simulation. First-person perspectives, by offering an embodied viewing experience, more directly activate self-projection mechanisms. Their spatial structure and subjective visual field closely align with viewers’ own perceptual frameworks, enabling the generation of more concrete situational experiences and action rehearsals (Zhang et al., 2023). The manner in which people are depicted in images also serves as a key social cue. Levels of concreteness, interactivity, and emotional expression significantly enhance perceived situational realism and psychological immersion (Zheng et al., 2022). In this process, visual cues function not merely as carriers of information but as active triggers of cognitive engagement and simulation generation.

Emotional states elicited during mental simulation further play an important role in shaping users’ attitudes and decision-making. Under conditions of incomplete information or limited cognitive resources, emotional experiences often serve as heuristic cues that influence judgments regarding information credibility, value, and expected outcomes (Jayawardena et al., 2023). Correspondingly, the coupling of emotional activation and motivational states strengthens individuals’ psychological connectedness to situational content and increases the likelihood of proactive engagement behaviors (Kumar et al., 2023). In the context of sports spectating, the content structure of UGC images can also evoke vicarious experiences. When images contain explicit human actions, interactive behaviors, and emotional expressions, viewers may experience a strong sense of presence and motivational readiness through mental simulation, even without direct physical participation in the event (Zhu et al., 2023). Such vicarious experiences initiate positive anticipatory states prior to actual behavior, thereby facilitating the formation of stable spectating intentions and behavioral tendencies.

Taken together, to more fully understand how social media user-generated content influences spectating intentions, this study integrates the self-referencing effect and mental simulation theory as its core theoretical foundations. The self-referencing effect suggests that individuals tend to allocate greater attention to information that is relevant to their own experiences or identities, thereby deepening comprehension and eliciting associative and emotional responses. Within UGC-based visual communication, human presence constitutes a socially meaningful visual element that can readily evoke memories of past experiences or prompt imagination of future situations, fostering a stronger sense of personal involvement. Meanwhile, mental simulation theory emphasizes that when individuals are exposed to concrete and vivid perceptual cues, they naturally construct internal images in which they imagine themselves acting, feeling, and responding within the depicted scenario. This imaginative activity not only increases psychological proximity to the visual content but also facilitates the formation of attitudes and behavioral tendencies.

From this perspective, self-referencing can be viewed as the initial trigger of mental simulation, while mental simulation renders self-referencing more concrete and elaborated. The two processes are closely interconnected and jointly promote a transition from passive information reception to active association, imagination, and emotional engagement when viewers encounter UGC images. Building on this psychological framework, the present research examines how the manner in which people are depicted in UGC images shapes viewers’ imaginative experiences and, in turn, their willingness to engage in spectating. Although prior studies have examined the influence of visual content on behavioral tendencies in domains such as tourism, advertising, and social communication (Huang and Ha, 2021; Wu and Lai, 2023; Lyu and Huang, 2024), systematic investigation remains limited in the specific context of sports spectating, particularly with respect to whether and how human presence appears in authentic user-uploaded images and what types of stadium scenes are depicted. Accordingly, this study focuses on two core elements of UGC images: whether people are present in the image and whether the image portrays a fan-oriented scene or a competition-oriented scene. By integrating self-referencing and mental simulation perspectives, the study seeks to reveal how different image configurations influence viewers’ psychological processes and behavioral tendencies. In doing so, it not only extends the application of mental simulation theory within sports communication research but also provides theoretical grounding and strategic insights for event marketing and social media content design.

### 2.3 User-generated content and user behavior

As a product of the Web 2.0 era, user-generated content has become one of the most influential forms of information dissemination in contemporary online environments. UGC refers to content created and shared by ordinary users through social media platforms, including text, images, and videos (Santos, 2022). A defining characteristic of UGC lies in its non-professional nature, which often renders it more authentic and credible in the eyes of audiences. As a result, an increasing number of brands, platforms, and marketers have turned to UGC as a means of attracting attention and stimulating user behavior. In the context of sports, UGC captures moments of sporting events in ways that not only enhance event visibility but also foster emotional connections with audiences, thereby motivating engagement and behavioral intentions.

The influence of UGC on consumer behavior, particularly in the domains of tourism and sports spectating, has received extensive scholarly attention. A substantial body of research demonstrates that UGC plays a critical role in audiences’ decision-making processes. Specifically, by providing emotional resonance and social validation, UGC reduces perceived uncertainty during decision making and facilitates the formation of behavioral intentions. In the tourism industry, travelers frequently share their experiences on social media platforms, and such content helps potential tourists construct perceptions and expectations regarding destinations (Wang et al., 2021). Similarly, in the context of sports events, audiences often participate in event dissemination by posting stadium photos and sharing spectating experiences on social media. These forms of UGC not only offer real-time feedback about events but also enhance potential spectators’ willingness to engage through emotional transmission and social interaction.

Within social media environments, the dissemination and interactive characteristics of UGC—particularly at the visual level—can significantly shape audiences’ behavioral and emotional responses. Among various visual elements, human figures constitute highly social and affectively oriented cues that are particularly effective in eliciting mental simulation processes. Through emotional resonance with others’ experiences, such processes prompt viewers to reconstruct situational scenarios and anticipate future behaviors (Sykora et al., 2022). Prior research indicates that the presence of people in images not only increases informational appeal but also deepens viewers’ sense of immersion and emotional identification with visual content (Ray and Bala, 2021). For example, destination images containing people are more likely to attract attention and elicit emotional responses in tourism advertising contexts (Cheung et al., 2022). Similar mechanisms are likely to operate in sports-related UGC, especially when stadium photos depict people who are perceived as relatable, thereby facilitating self-involvement and mental simulation and ultimately enhancing spectating motivation and behavioral intentions.

However, existing findings regarding the effects of human presence in UGC images remain inconclusive. Some studies suggest that the inclusion of people significantly strengthens emotional connection and behavioral intentions (Li and Wan, 2025), whereas others report that human presence does not exert a significant influence on user behavior in certain contexts (Li and Xie, 2020). These inconsistencies may stem from variations in image context, modes of human depiction, and audience characteristics, including cultural background. Accordingly, a more systematic examination of the mechanisms through which human presence operates across different contexts is warranted.

In sports-related UGC images, characteristics of stadium context further condition how human presence influences spectators’ behavioral intentions. Stadium contexts can generally be categorized into two types: fan-oriented contexts and competition-oriented contexts. Fan-oriented contexts emphasize social interaction and a sense of belonging, whereas competition-oriented contexts focus on athletic performance and competitive dynamics. Prior research indicates that human figures in fan-oriented contexts are more likely to evoke social identification and feelings of belonging, thereby increasing spectators’ willingness to watch events, whereas human figures in competition-oriented contexts primarily attract attention to performance and gameplay processes (Plank, 2016). These context-dependent effects underscore the interactive relationship between stadium context and human presence, a mechanism that has yet to be fully explored.

Taken together, the dissemination effects of UGC depend not only on content quality but also on a combination of visual characteristics, human presence, and contextual cues embedded in images. Although prior research has examined the role of UGC in tourism and advertising contexts, systematic investigation of the interactive effects of human presence and stadium context remains limited in the domain of sports events. Future research should further examine the complex relationships among human depiction, stadium context, and audience behavior, thereby providing deeper theoretical insight and practical guidance for sports communication and marketing strategies on social media platforms.

### 2.4 Human presence in stadium photos and spectating intention

Prior research in consumer psychology has demonstrated that incorporating human elements into visual product descriptions can effectively activate consumers’ prior situational memories of interacting with products. The core mechanism underlying this effect lies in individuals’ ability to mentally involve their own bodies in interactions with products or situations through depicted human figures, thereby forming strong and concrete situational associations. Specifically, when consumers encounter images containing people, these human depictions function as cognitive triggers that facilitate the retrieval and activation of previous interaction experiences, prompting consumers to mentally construct vivid scenarios of engaging with the product or situation (D’Argembeau and Mathy, 2011). For example, studies show that real human models wearing apparel enable consumers to more vividly imagine themselves wearing the clothing compared with virtual models, thereby increasing purchase intentions (Boardman and Mccormick, 2019; Bagatini et al., 2023). Such mental simulation elicited by human presence strengthens emotional responses and behavioral motivation.

From perspectives in human behavior and social psychology, the presence of people in visual scenes not only serves as a social signal but also activates emotional and psychological connections that stimulate situational simulation of future behaviors. Particularly in tourism and consumption contexts, research indicates that images containing people allow viewers to more easily project themselves into the depicted environment and experience its atmosphere and emotional tone (Karl et al., 2021). This suggests that human presence facilitates vivid mental representations of future situations, thereby enhancing engagement intentions and behavioral motivation. In tourism imagery, viewers are able to construct a psychological experience of “my role in the scene,” which in turn increases their willingness to participate and act.

In the context of sports events, the visual characteristics of stadium photos differ from those of tourism and advertising images. Sports events are marked by strong dynamics, competition, and social interaction, rendering human presence not merely a vehicle for information transmission but a critical trigger of emotional connection and mental simulation between spectators and events. Unlike static depictions of people in tourism or product advertising, human figures in sports imagery often convey athletes’ competitive states, emotional reactions, or interactions with spectators. These dynamic visual elements can evoke a sense of presence and vicarious experience among viewers (Kim et al., 2023). For instance, when viewers encounter stadium photos depicting athletes engaged in intense competition or fans enthusiastically interacting, they may imagine themselves participating in the scene, forming situational reenactments in which they assume the role of spectator or even athlete. This mental simulation process enhances their motivation to engage with the event.

Human presence in stadium photos not only elicits emotional resonance but also increases behavioral intentions by strengthening mental simulation. Visual elements such as athletes’ movements, fans’ interactions, and the competitive context enable viewers to mentally place themselves within the scene, allowing them to experience the tension and excitement of the event. This sense of immersion and situational reenactment constitutes a key mechanism through which sports visual content establishes emotional connections with audiences and stimulates behavioral motivation. Moreover, the unique emotional and social characteristics of sports events distinguish the influence mechanisms of stadium photos from those observed in other domains (Rai et al., 2025). In sports contexts, human presence not only triggers emotional responses but also enhances social identification. Similar to the effects observed in tourism imagery, depictions of people in sports events strengthen spectators’ sense of belonging and participation, thereby increasing spectating intentions (Chalip et al., 2003; Lim et al., 2015). Unlike static tourism scenes, however, human presence in sports imagery additionally involves mental simulation of competitive processes and anticipation of future viewing behavior, which further amplifies spectators’ motivation to engage (Chang et al., 2021).

Based on this theoretical background and existing empirical evidence, human presence in stadium photos is expected to effectively activate spectators’ mental simulation, enhance emotional connection, and ultimately increase spectating intention. In sports events characterized by high emotional intensity and social interaction, human figures function not only as triggers of affective linkage but also as critical facilitators of mental simulation. Accordingly, the following hypotheses are proposed.

*H1*: Compared with stadium photos that do not contain human figures, stadium photos featuring human presence are more likely to enhance users’ spectating intention.

*H2*: Mental simulation mediates the effect of human presence in stadium photos on observers’ spectating intention. Specifically, stadium photos featuring human presence (vs. no human presence) are more likely to elicit observers’ mental simulation, which in turn increases their spectating intention.

### 2.5 The moderating role of stadium context in sports photos

Prior research has widely acknowledged that situational factors play a critical role in shaping individuals’ cognition and behavior, particularly in contexts involving emotional responses and social interaction (Lopes et al., 2005). Empirical evidence suggests that the social and affective characteristics of a given context are central to the formation of behavioral intentions (Lee et al., 2019). Within sports event environments, the characteristics of the stadium context exert a pronounced moderating influence on spectators’ emotional reactions and motivational states (Guo et al., 2024). Specifically, stadium context shapes how spectators interpret human presence in stadium photos and subsequently activates mental simulation processes, thereby influencing spectating intention.

First, existing studies indicate that the intensity of social interaction embedded in a context affects the degree of individuals’ emotional engagement. In fan-oriented contexts, interaction among spectators and a sense of community belonging are often amplified. Stadium photos captured in such contexts not only depict athletes’ performance but also emphasize interactions among fans and shared experiential moments. Scenes such as collective cheering, group celebrations, and emotional resonance enable viewers to perceive stronger social connections (Lai et al., 2022). When human presence is embedded within fan-oriented contexts, spectators are more likely to project themselves into scenarios of jointly enjoying the event with others, which elicits stronger emotional responses and mental simulation. This heightened sense of immersion and affective resonance increases spectators’ interest in the event and fosters stronger behavioral motivation and spectating intention.

In contrast, competition-oriented contexts emphasize athletic performance, competitive tension, and confrontation between athletes. Although such contexts are effective in capturing attention, they typically involve limited social interaction and emotional exchange among spectators. Stadium photos in competition-oriented contexts primarily highlight athletes’ intense competition and the inherent tension of the event, directing viewers’ focus toward performance rather than social engagement. Under these conditions, while human presence may still trigger a certain degree of mental simulation, the absence of rich social interaction and shared emotional experience constrains emotional involvement (Jiang et al., 2019). Consequently, the effect of human presence on spectating intention tends to be weaker in competition-oriented contexts.

Moreover, social context and emotional connection play a central role in motivating sports consumption. Prior research demonstrates that sociality and emotional resonance significantly enhance individuals’ engagement motivation and behavioral intention (Wu et al., 2013). In fan-oriented contexts, human presence in stadium photos conveys stronger cues of social interaction and collective emotion, which more effectively activate spectators’ mental simulation. Through this process, viewers are more likely to mentally construct scenarios in which they themselves participate in the event. Emotional resonance and perceived social interaction jointly facilitate psychological immersion, thereby increasing spectating intention.

Based on the above reasoning, this study proposes the following hypothesis.

*H3*: Stadium context (fan-oriented context vs competition-oriented context) moderates the effect of human presence on spectating intention. Specifically, in fan-oriented contexts, which emphasize spectator interaction, community belonging, and emotional resonance, human presence in stadium photos is more likely to interact with contextual cues to activate mental simulation and subsequently enhance spectating intention.

**Figure 1:**
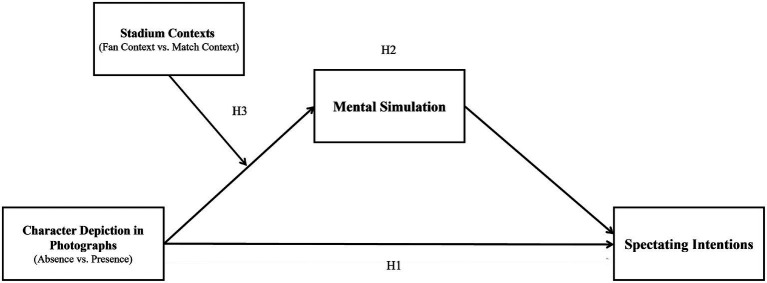
Conceptual framework of the proposed research model.

Figure 1 presents the conceptual framework of this study and summarizes the proposed hypotheses.

## 3 Study 1

### 3.1 Methods

#### 3.1.1 Participants and experimental design

Study 1 aimed to examine how human presence in stadium photos influences users’ spectating intentions. Study 1 employed a one-factor, two-level between-subjects design, with human presence manipulated as present versus absent. Participants were recruited through the Credamo platform, https://www.credamo.com, and completed the online experimental task. To improve participation and data quality, an appropriate incentive mechanism was used, and eligible participants received monetary compensation after completing the task. A total of 166 participants were initially recruited. Recruitment advertisements were posted on Credamo, and strict screening criteria were applied during data collection. Specifically, participants who completed the questionnaire in less than 60 s or failed the attention check were excluded to ensure data quality and validity. Finally, 148 valid participants were included in the analysis. Among them, 58.11% were male and 41.89% were female. In terms of age distribution, 77.70% of the participants were between 31 and 40 years old. Regarding educational background, 47.30% held a bachelor’s degree. In terms of monthly income, participants earning 3,000 to 5,999 RMB and those earning 6,000 to 8,999 RMB accounted for the same proportion, both at 41.89%. The demographic information for Study 1 is presented in Table 1.

**Table 1:** Demographic information.

Demographic variables	Frequency (N)	Percentage (%)
Study 1		
Gender		
Male	86	58.11%
Female	62	41.89%
Age		
Under 20	0	0.00%
21–30	10	6.76%
31–40	115	77.70%
41–50	23	15.54%
51–60	0	0.00%
Over 60	0	0.00%
Educational background		
High school and below	0	0.00%
High school/Technical school	18	12.16%
Junior college	55	37.16%
Bachelor’s degree	70	47.30%
Master’s degree	5	3.38%
Doctoral degree	0	0.00%
Monthly income (RMB)		
Less than 3,000	4	2.70%
3,000–5,999	62	41.89%
6,000–8,999	62	41.89%
9,000–11,999	14	9.46%
12,000 and above	6	4.05%
Study 2		
Gender		
Male	90	60.00%
Female	60	40.00%
Age		
Under 20	0	0.00%
21–30	15	10.00%
31–40	120	80.00%
41–50	10	6.67%
51–60	5	3.33%
Over 60	0	0.00%
Educational background		
High school and below	0	0.00%
High school/Technical school	5	3.33%
Junior college	55	36.67%
Bachelor’s degree	72	48.00%
Master’s degree	18	12.00%
Doctoral degree	0	0.00%
Monthly income (RMB)		
Less than 3,000	5	3.33%
3,000–5,999	50	33.33%
6,000–8,999	63	42.00%
9,000–11,999	18	12.00%
12,000 and above	14	9.33%
Study 3		
Gender		
Male	91	59.87%
Female	61	40.13%
Age		
Under 20	0	0.00%
21–30	12	7.89%
31–40	122	80.26%
41–50	14	9.21%
51–60	3	1.97%
Over 60	1	0.66%
Educational background		
High school and below	0	0.00%
High school/Technical school	6	3.95%
Junior college	56	36.84%
Bachelor’s degree	73	48.00%
Master’s degree	16	10.53%
Doctoral degree	1	0.66%
Monthly income (RMB)		
Less than 3,000	7	4.61%
3,000–5,999	52	34.21%
6,000–8,999	64	42.00%
9,000–11,999	16	10.53%
12,000 and above	13	8.55%

#### 3.1.2 Stimulus materials

**Figure 2:**
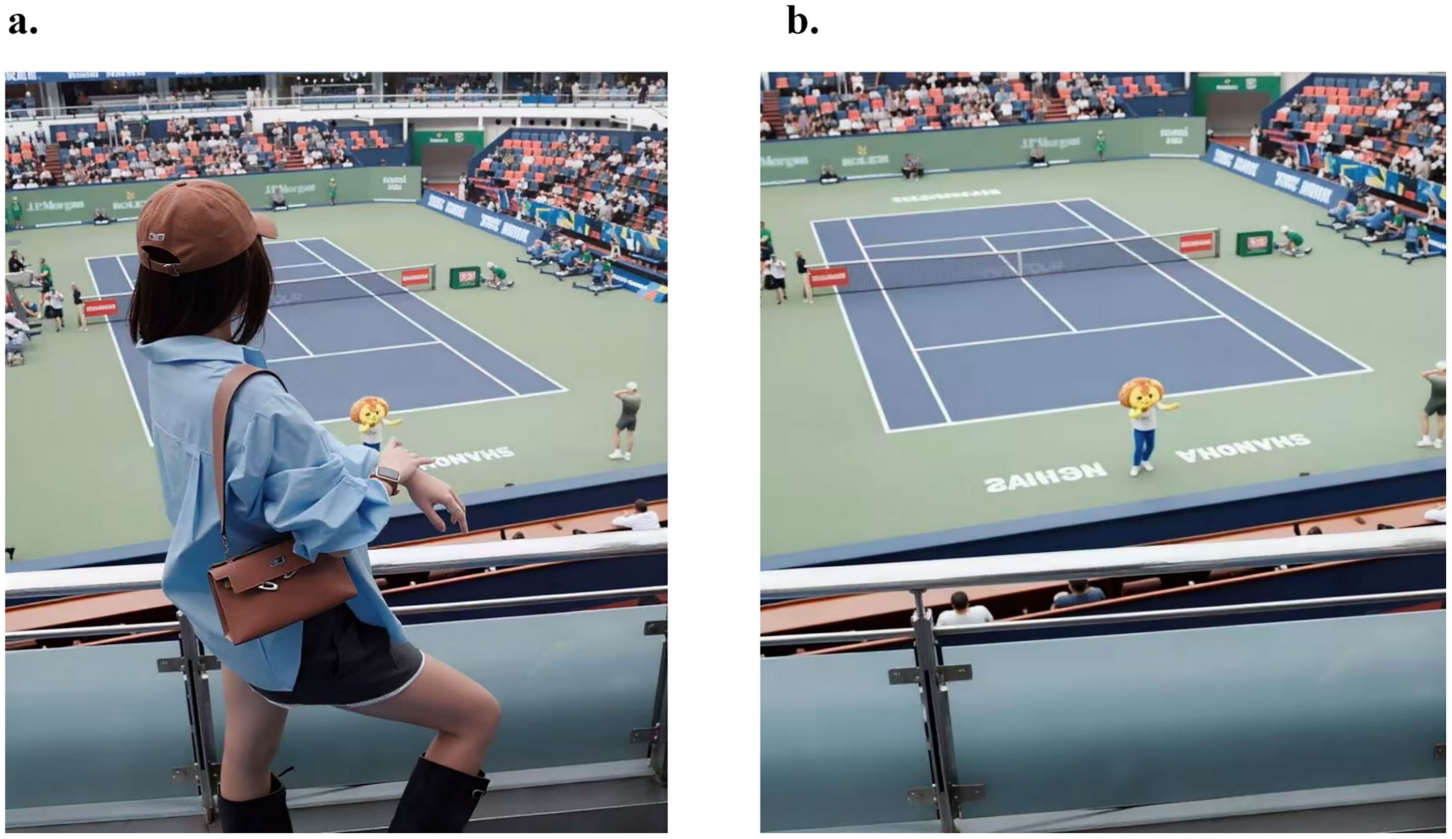
Photo stimuli used in Study 1. **(a)** Shows a stadium photo containing one non-face-revealing UGC creator. **(b)** Shows a stadium photo without a UGC creator.

The stimuli used in this experiment consisted of two sets of stadium photos. One set contained one non-face-revealing UGC creator in the stadium scene, whereas the other set did not contain any UGC creator. All photos were selected from representative sports events to ensure the authenticity and experiential quality of the scenes. In the human-presence condition, the photo included one UGC creator whose face was not shown, and only the interaction with the competition context or emotional expression was presented. In the no-human-presence condition, the photo displayed only the stadium and related facilities, without any human elements, as shown in Figure 2. The photos were carefully screened to ensure consistency in style, composition, lighting, and other visual features across the two conditions, thereby reducing potential interference from extraneous variables.

All stimuli were standardized using professional image-editing software to ensure consistency in image quality. To further verify the suitability of the stimuli, participants evaluated the perceived authenticity of the photos before the formal experiment. Based on the pretest feedback, images with relatively high acceptance in terms of photo content and human-presence manipulation were selected, ensuring the validity of the experiment and participants’ sense of immersion.

#### 3.1.3 Procedure and measures

Participants in Study 1 were recruited through the Credamo platform and completed an online experimental task. At the beginning of the experiment, all participants first read a brief study instruction and provided informed consent. They were then randomly assigned to one of two experimental conditions, namely the human-presence condition, *N* = 74, and the no-human-presence condition, *N* = 74. Each participant viewed the stadium photo for 3 min and was instructed to pay close attention to the details in the photo. During the experiment, participants were asked to imagine themselves being present in the scene in order to activate their mental simulation of the stadium experience.

The main dependent variable in this study was spectating intention. Spectating intention was measured using a six-item scale assessing participants’ interest, intention, and anticipated behavior regarding watching sports events, adapted from Cho et al. (2019). The items were as follows: I plan to attend a live sports event in the near future, I would like to attend this sports event in person, I think watching this event would make me feel happy, I would be willing to share my experience of watching this event with others, I would recommend this event to my friends, and I would encourage my friends to watch this event. All items were rated on a seven-point Likert scale, ranging from 1, strongly disagree, to 7, strongly agree. The reliability of the scale was acceptable, Cronbach’s *α* = 0.799, indicating adequate internal consistency.

To verify the effectiveness of the experimental manipulation, a manipulation check item was included to assess whether participants noticed the human element in the photo and how strongly they perceived the presence of the person. This item was used to examine whether the human-presence manipulation was successful and whether the experimental conditions were perceived as intended. The specific item was: To what extent do you perceive the presence of the UGC creator in the photo. Responses were rated from 1, no perceived human presence at all, to 7, extremely obvious human presence. In addition, participants rated the perceived authenticity of the photo as a manipulation-related control item to verify the effectiveness of the image manipulation. Finally, all participants reported basic demographic information, including gender, age, and income, which was used in subsequent analyses.

On the basis of the manipulation check and authenticity control, this study further measured two non-target visual attributes, namely photo clarity and image attractiveness. Photo clarity was measured to examine whether the two sets of photos were consistent in visual recognizability and ease of information processing, Cronbach’s α = 0.843. The items included: This photo is clear, The main visual elements in this photo are easy to recognize, and This photo is visually easy to view. This measure was adapted from research on visual information quality and image-based information presentation (Kim and Lennon, 2000). Image attractiveness was measured to examine whether the two sets of photos were consistent in overall aesthetic evaluation and visual appeal, Cronbach’s α = 0.811. The items included: This photo is visually attractive, This photo is esthetically pleasing, and The overall visual presentation of this photo is good. This measure was adapted from research on perceived aesthetic quality and visual attractiveness (Lavie and Tractinsky, 2004). Finally, participants provided demographic information, including gender, age, educational background, and monthly income.

### 3.2 Results

#### 3.2.1 Manipulation check

This study first examined whether the manipulation of human presence in the stadium photos was effective. An independent-samples *t*-test was conducted to compare perceived human presence between the human-presence condition and the no-human-presence condition. The results showed that participants in the human-presence condition reported significantly higher perceived human presence than those in the no-human-presence condition (*M* = 5.189, SD = 1.863 vs. *M* = 2.743, SD = 1.752, *t*(146) = −8.227, *p* < 0.001). This result indicates that the human-presence manipulation was successful.

In addition, to ensure that human presence in the stadium photos was not confounded with other variables, this study examined whether the two conditions differed in perceived photo authenticity. The independent-samples *t*-test showed no significant difference in perceived photo authenticity between the human-presence and no-human-presence conditions (*M* = 5.445, SD = 1.356 vs. *M* = 5.419, SD = 1.365, *t*(146) = −0.121, *p* = 0.904). This result suggests that photo authenticity was effectively controlled across the two conditions.

On this basis, this study further examined whether the two sets of photos differed in photo clarity and image attractiveness. The results showed no significant difference in photo clarity between the human-presence and no-human-presence conditions (*M* = 5.561, SD = 1.041 vs. *M* = 5.493, SD = 1.083, *t*(146) = −0.389, *p* = 0.698). This indicates that the photos with and without human presence did not systematically differ in visual clarity or recognizability of the main visual elements. In addition, the two conditions did not significantly differ in image attractiveness (*M* = 5.284, SD = 1.145 vs. *M* = 5.203, SD = 1.174, *t*(146) = −0.425, *p* = 0.672). This result suggests that the two sets of photos were comparable in overall visual clarity and visual appeal.

Together, these results indicate that the human-presence manipulation in Study 1 was successful, while the two sets of photos did not significantly differ in non-target visual attributes, including authenticity, clarity, and image attractiveness. Therefore, the subsequent differences in spectating intention are unlikely to be driven by differences in photo authenticity, photo clarity, or overall visual attractiveness, but are more likely attributable to the core manipulation of whether a person was present in the stadium photo.

#### 3.2.2 Main effect analysis

**Figure 3:**
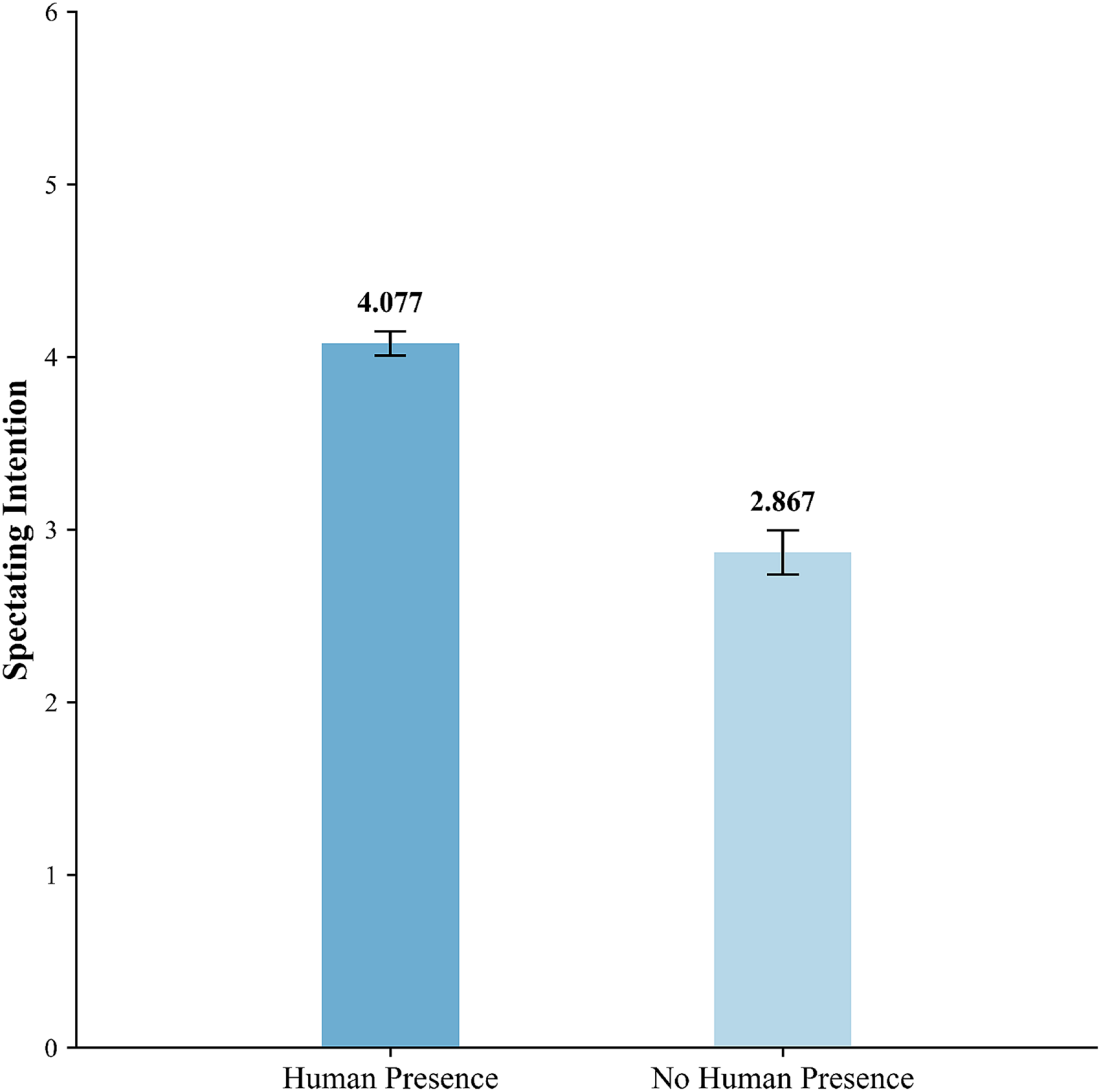
Result of Study 1.

Human presence in stadium photos was entered as the independent variable, and spectating intention was treated as the dependent variable. The results indicated that participants exposed to stadium photos with human presence reported significantly higher spectating intention (*M* = 4.077, SD = 0.604) than those exposed to photos without human presence (*M* = 2.867, SD = 1.102, *t*(146) = −8.281, *p* < 0.001). These findings demonstrate that human presence in stadium photos significantly enhances spectating intention (Seeing Figure 3). Therefore, *H1* is supported.

#### 3.2.3 Supplementary robustness analysis controlling for demographic variables

Considering that the age distribution of participants in Study 1 was relatively concentrated, this study further conducted a supplementary robustness analysis using a general linear model to examine whether the effect of human presence on spectating intention remained robust after controlling for demographic variables. Specifically, spectating intention was entered as the dependent variable, human presence was entered as the fixed factor, and age, gender, education, and income were included as categorical control variables. The results showed that, after controlling for these demographic variables, the effect of human presence on spectating intention remained significant (*F*(1, 137) = 66.343, *p* < 0.001). This result indicates that the main effect in Study 1 remained robust after controlling for demographic characteristics, and that the difference in spectating intention was unlikely to be driven by differences in age, gender, educational background, or income.

## 4 Study 2

### 4.1 Methods

#### 4.1.1 Participants and experimental design

Study 2 aimed to examine how human presence in stadium photos influences users’ spectating intention. Study 2 employed a single-factor, two-level between-subjects design, with human presence manipulated as present versus absent. Participants were recruited through the Credamo platform, https://www.credamo.com, and completed the online experimental task. To improve participation and data quality, an appropriate incentive mechanism was used, and eligible participants received monetary compensation after completing the task. A total of 170 participants were initially recruited. Recruitment advertisements were posted on Credamo, and strict screening criteria were applied during data collection. Specifically, participants who completed the questionnaire in less than 60 s or failed the attention check were excluded to ensure data quality and validity. Finally, 150 valid participants were included in the analysis. Among them, 60.00% were male and 40.00% were female. In terms of age distribution, 80.00% of the participants were between 31 and 40 years old. Regarding educational background, 48.00% held a bachelor’s degree. In terms of monthly income, 42.00% of the participants reported a monthly income between 6,000 and 8,999 RMB. The demographic information for Study 2 is presented in Table 1.

#### 4.1.2 Stimulus materials

**Figure 4:**
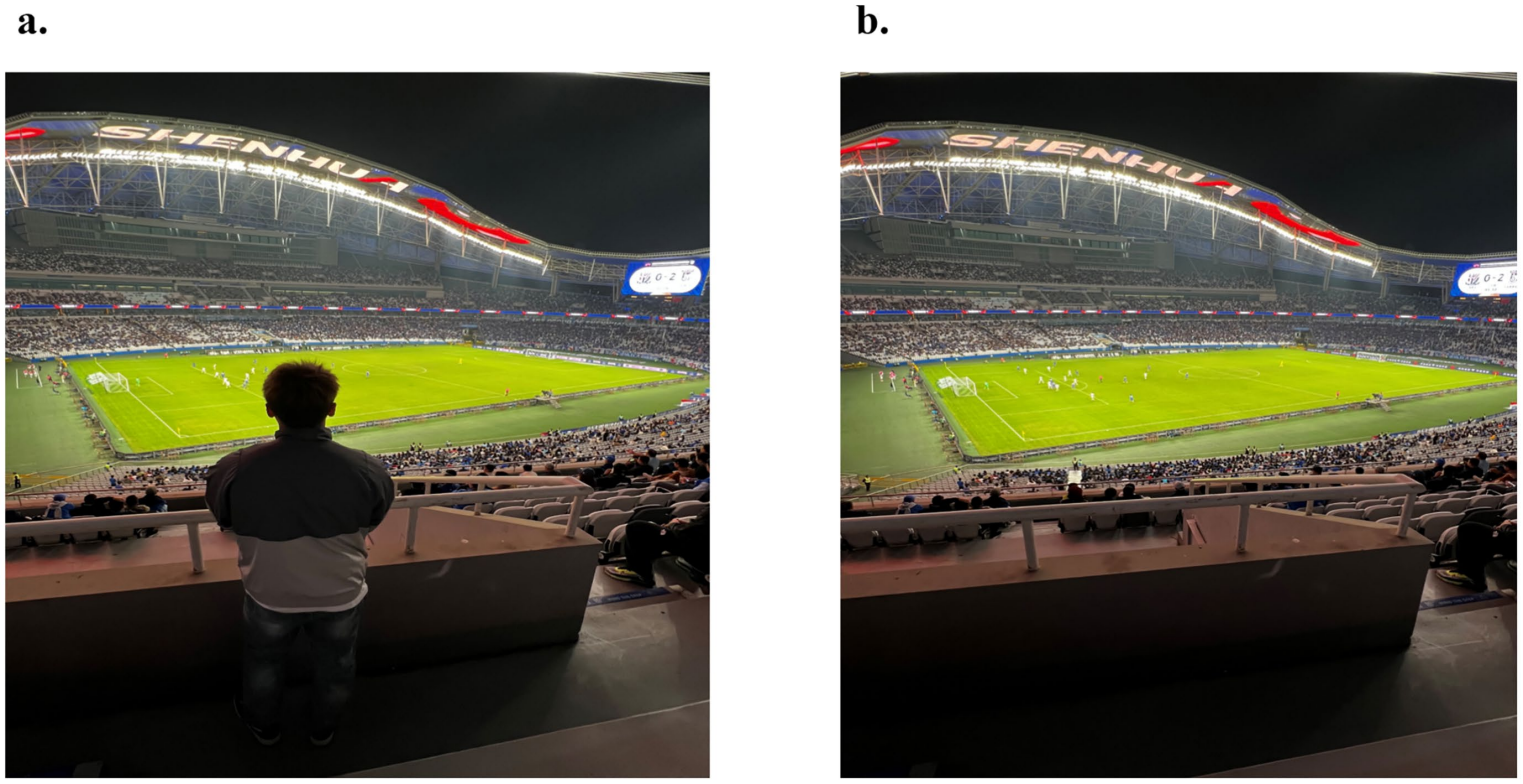
Photo stimuli used in Study 2. **(a)** Shows a stadium photo containing one non-face-revealing UGC creator. **(b)** Shows a stadium photo without a UGC creator.

The stimulus materials used in this experiment consisted of two sets of stadium photos. One set contained one non-face-revealing UGC creator in the stadium scene, whereas the other set did not contain any human figure. All photos were selected from representative sports events to ensure the authenticity and experiential quality of the scenes. In the human-presence condition, the photo included one UGC creator whose face was not shown, and only the creator’s interaction with the competition context or emotional expression was presented. In the no-human-presence condition, the photo displayed only the stadium and related facilities, without any human elements, as shown in Figure 4. The photos were carefully screened to ensure consistency in style, composition, lighting, and other visual features across the two conditions, thereby reducing potential interference from extraneous variables.

All stimuli were standardized using professional image-editing software to ensure consistency in image quality. To further verify the suitability of the stimuli, participants evaluated the perceived authenticity of the photos before the formal experiment. Based on the pretest feedback, images with relatively high acceptance in terms of photo content and human-presence manipulation were selected, ensuring the validity of the experiment and participants’ sense of immersion.

#### 4.1.3 Procedure and measures

Participants in Study 2 were recruited through the Credamo platform and completed the online experimental task. At the beginning of the experiment, all participants first read a brief study introduction and provided informed consent. They were then randomly assigned to one of two experimental conditions, namely the human-presence condition (*N* = 75) or the no-human-presence condition (*N* = 75). Each participant viewed the stadium photo for 3 min and was instructed to pay close attention to the details in the photo. During the experiment, participants were asked to imagine themselves being physically present in the scene in order to activate their mental simulation of the stadium experience.

This study mainly measured participants’ spectating intention and mental simulation. Spectating intention was assessed using a six-item scale covering participants’ interest, intention, and anticipated behavior related to watching sports events. The items were as follows: I plan to attend a live sports event in the near future; I would like to attend this sports event in person; I think watching this event would make me feel happy; I would be willing to share my experience of watching this event with others; I would recommend this event to my friends; and I would encourage my friends to watch this event. All items were rated on a seven-point Likert scale ranging from 1, strongly disagree, to 7, strongly agree. The scale showed good reliability (Cronbach’s *α* = 0.847), indicating adequate internal consistency.

Mental simulation was measured using a three-item scale designed to assess participants’ affective and cognitive imagery after viewing the stadium photo. The items were as follows: I can imagine myself being physically present while watching this match; I can mentally construct a scene of myself watching the match; and I can feel the atmosphere of the stadium. These items were also rated on a seven-point Likert scale ranging from 1, strongly disagree, to 7, strongly agree. The scale showed high internal consistency (Cronbach’s α = 0.881).

To ensure the effectiveness of the experimental manipulation, a manipulation check item was included to examine whether participants noticed the human element in the photo and how strongly they perceived the manner of human presence. This item was used to verify whether the human-presence manipulation was successful and whether the experimental conditions were perceived as intended. The specific item was: To what extent do you perceive the presence of the UGC creator in the photo. Responses were rated from 1, no perceived human presence at all, to 7, extremely obvious human presence. In addition, participants rated the perceived authenticity of the photo as a manipulation-related control item to verify the effectiveness of the image manipulation. On the basis of the manipulation check and authenticity control, this study further measured two non-target visual attributes, namely photo clarity (Cronbach’s α = 0.765) and image attractiveness (Cronbach’s α = 0.878). Finally, all participants reported basic demographic information, including gender, age, and income, which was used in subsequent analyses.

### 4.2 Results

#### 4.2.1 Manipulation check

This study first examined whether the manipulation of human presence in the stadium photos was effective. An independent-samples *t*-test was conducted to compare perceived human presence between the human-presence condition and the no-human-presence condition. The results showed that participants in the human-presence condition reported significantly higher perceived human presence than those in the no-human-presence condition (*M* = 5.626, SD = 1.239 vs. *M* = 2.920, SD = 1.421, *t*(148) = −12.432, *p* < 0.001). This result indicates that the human-presence manipulation was successful.

In addition, to ensure that human presence in the stadium photos was not confounded with other variables, such as photo quality, this study examined whether the two conditions differed in perceived photo quality. The independent-samples *t*-test showed no significant difference in perceived photo quality between the human-presence and no-human-presence conditions (*M* = 5.760, SD = 1.125 vs. *M* = 5.653, SD = 0.814, *t*(148) = −0.665, *p* = 0.507). This result suggests that photo quality was effectively controlled across the two conditions.

On this basis, this study further examined whether the two sets of photos differed in photo clarity and image attractiveness. The results showed no significant difference in photo clarity between the human-presence and no-human-presence conditions (*M* = 5.587, SD = 0.914 vs. *M* = 5.493, SD = 0.947, *t*(148) = −0.618, *p* = 0.538). This indicates that the photos with and without human presence did not systematically differ in visual clarity, recognizability of the main visual elements, or ease of information processing. In addition, the two conditions did not significantly differ in image attractiveness (*M* = 5.341, SD = 1.008 vs. *M* = 5.227, SD = 1.052, *t*(148) = −0.679, *p* = 0.498). This result suggests that the two sets of photos were comparable in overall visual appeal and aesthetic evaluation.

#### 4.2.2 Main effect analyses

Human presence in stadium photos was entered as the independent variable and spectating intention as the dependent variable. The results indicated that participants exposed to stadium photos with human presence reported significantly higher spectating intention (*M* = 5.187, SD = 0.984) than those exposed to photos without human presence (*M* = 3.696, SD = 0.952), *t*(148) = −9.429, *p* < 0.001. This finding provides further evidence that human presence in stadium photos significantly enhances spectating intention.

**Figure 5:**
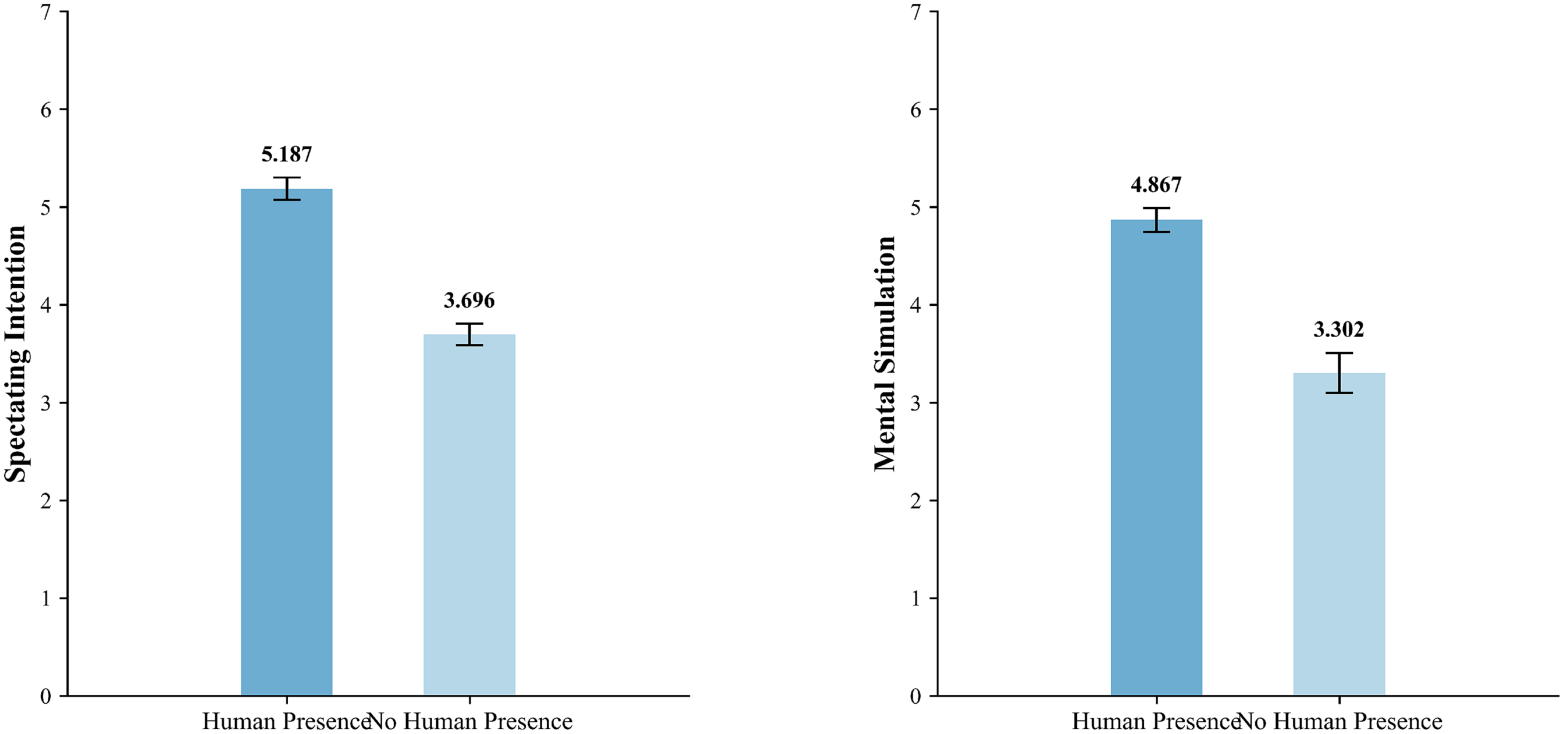
Result of Study 2.

Human presence was also entered as the independent variable and mental simulation as the dependent variable. The results showed that participants in the human presence condition reported significantly higher levels of mental simulation (*M* = 4.867, SD = 1.051) than those in the no human presence condition (*M* = 3.302, SD = 1.764), *t*(148) = −6.598, *p* < 0.001. This result indicates that human presence in stadium photos effectively increases viewers’ mental simulation (Seeing Figure 5).

#### 4.2.3 Mediation analysis

To examine the mechanism through which human presence in stadium photos influences users’ spectating intention, a bias-corrected bootstrapping mediation analysis was conducted using SPSS PROCESS Model 4 with 5,000 resamples. This analysis tested the mediating role of mental simulation in the relationship between human presence in stadium photos and spectating intention (Hayes and Preacher, 2014). The results showed that human presence in stadium photos had a significant direct effect on users’ spectating intention (*β* = 1.491, LLCI = 1.177, ULCI = 1.804, excluding zero). In addition, mental simulation exerted a significant mediating effect in the relationship between human presence and spectating intention (*β* = 0.7425, LLCI = 0.183, ULCI = 1.179, excluding zero). These findings indicate that human presence enhances users’ spectating intention by activating viewers’ mental simulation. Therefore, *H2* is supported.

#### 4.2.4 Supplementary robustness analysis controlling for demographic variables

Considering that the age distribution of participants in Study 2 was relatively concentrated, age, gender, education, and income were further included as control variables in the PROCESS Model 4 mediation model. A bootstrapping procedure with 5,000 resamples was used to test the indirect effect of mental simulation. The results showed that, after controlling for these demographic variables, the indirect effect of human presence on spectating intention through mental simulation remained significant (*β* = 0.739, BootSE = 0.181, 95% CI [0.421, 1.112], excluding zero). This result indicates that the mediating effect of mental simulation in Study 2 remained robust after controlling for age, gender, educational background, and income, providing further support for *H2*.

## 5 Study 3

### 5.1 Methods

#### 5.1.1 Participants and experimental design

Study 3 aimed to examine how human presence in stadium photos and stadium context jointly influence users’ spectating intention. Study 3 adopted a 2 (human presence: human presence vs. no human presence) × 2 (stadium context: fan-oriented context vs. competition-oriented context) between-subjects design. Accordingly, four experimental conditions were created, namely human presence in a fan-oriented context, human presence in a competition-oriented context, no human presence in a fan-oriented context, and no human presence in a competition-oriented context.

To improve participation and data quality, recruitment information was posted through the Credamo platform, https://www.credamo.com, and participants completed the online experimental task. Eligible participants received monetary compensation after completing the task. To ensure data quality, all participants were required to pass an attention check during screening, and questionnaires completed in less than 60 s were excluded. A total of 170 participants were initially recruited. After strict screening, 152 valid participants were retained for analysis. Among them, 59.87% were male and 40.13% were female. In terms of age distribution, 80.26% of the participants were between 31 and 40 years old. Regarding educational background, 48.00% held a bachelor’s degree. In terms of monthly income, 42.00% of the participants reported a monthly income between 6,000 and 8,999 RMB. The demographic information for Study 3 is presented in Table 1.

#### 5.1.2 Stimulus materials

The stimulus materials consisted of four sets of stadium photos: (1) stadium photos featuring one faceless UGC creator in a fan-oriented context, (2) stadium photos featuring one faceless UGC creator in a competition-oriented context, (3) stadium photos without a UGC creator in a fan-oriented context, and (4) stadium photos without a UGC creator in a competition-oriented context. All photos were selected from representative sports events to ensure realism and a sense of immersion.

**Figure 6:**
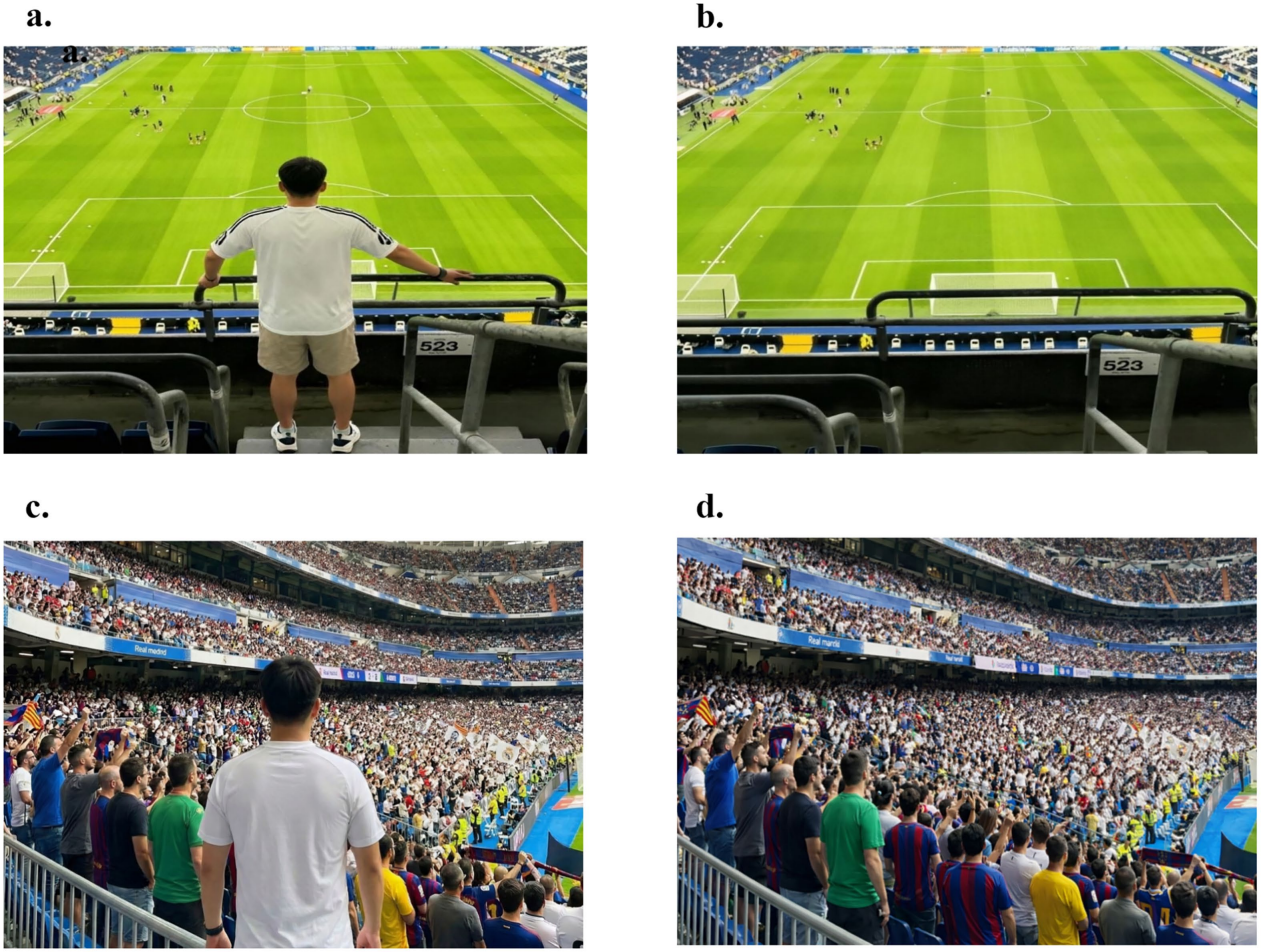
Photo stimuli used in Study 3. **(a)** Shows a stadium photo containing one non-face-revealing UGC creator in a competition-oriented context. **(b)** Shows a competition-oriented stadium photo without a UGC creator. **(c)** Shows a stadium photo containing one non-face-revealing UGC creator in a fan-oriented context. **(d)** Shows a fan-oriented stadium photo without a UGC creator.

In the human presence conditions, the individual depicted in the photo was a UGC creator whose face was not visible; only interactions with the stadium environment or emotional expressions related to the event were shown. In the no human presence conditions, the photos depicted only the stadium and related facilities, without any identifiable individuals (Seeing Figure 6).

Stadium context was manipulated as either a fan-oriented context or a competition-oriented context. Fan-oriented contexts emphasized audience interaction and emotional resonance, such as cheering and collective celebration, whereas competition-oriented contexts focused on the intensity of the match and athletic confrontation, highlighting players’ skills and the tension of the competition. To ensure consistency across conditions, all photos were carefully screened and standardized, with similar visual style, composition, and lighting.

#### 5.1.3 Procedure and measures

Participants in Study 3 were recruited through Credamo and completed the experiment online. At the beginning of the experiment, all participants read a brief introduction and provided informed consent. They were then randomly assigned to one of the four experimental conditions: human presence in a fan-oriented context (*N* = 38), human presence in a competition-oriented context (*N* = 38), no human presence in a fan-oriented context (*N* = 38), or no human presence in a competition-oriented context (*N* = 38).

Each participant viewed stadium photos for 2 min and was instructed to pay close attention to the details of the images. During viewing, participants were explicitly asked to imagine themselves as if they were physically present at the scene, in order to facilitate mental simulation.

Spectating intention and mental simulation were the primary dependent variables. Spectating intention was measured using a six-item scale assessing participants’ interest in watching sports events, behavioral intentions, and anticipated behaviors. Sample items included “I plan to attend a live sports event in the near future,” “I want to watch this match on site,” “I think watching this match would make me feel happy,” “I would like to share my experience of watching this match with others,” “I would recommend this event to my friends,” and “I would encourage my friends to attend this event.” All items were rated on seven-point Likert scales ranging from 1 (strongly disagree) to 7 (strongly agree). The scale demonstrated acceptable reliability (Cronbach’s *α* = 0.805).

Mental simulation was measured using three items assessing participants’ imagined emotional and cognitive experiences after viewing the stadium photos. The items included “I can imagine myself being at the stadium watching this match,” “I can mentally construct a scene of myself watching the match,” and “I can feel the atmosphere of the stadium.” These items were also rated on seven-point Likert scales (1 = strongly disagree, 7 = strongly agree), with high internal consistency (Cronbach’s α = 0.909).

To ensure the effectiveness of the experimental manipulation, a manipulation check item was included to examine whether participants noticed the human element in the photo and how strongly they perceived the human presence. The purpose of this item was to verify the effectiveness of the human-presence manipulation and to ensure that the experimental conditions were perceived as intended. The specific item was: To what extent do you perceive the presence of the UGC creator in the photo. Responses were rated from 1, no perceived human presence at all, to 7, extremely obvious human presence.

In addition, participants rated the perceived authenticity of the photo as a manipulation-related control item to verify the effectiveness of the image manipulation. On the basis of the manipulation check and authenticity control, this study further measured two non-target visual attributes, namely photo clarity (Cronbach’s α = 0.863) and image attractiveness (Cronbach’s α = 0.802). Finally, all participants reported basic demographic information, including gender, age, and income, which was used in subsequent analyses.

### 5.2 Results

#### 5.2.1 Manipulation checks

To ensure the effectiveness of the experimental manipulation, this study first examined whether the manipulation of human presence in the stadium photos was successful. In the manipulation check, participants were asked to evaluate the extent to which they perceived human presence in the photos. Specifically, participants responded to the item, To what extent do you perceive the presence of a person in the photo. The results showed that participants in the human-presence condition reported significantly higher perceived human presence than those in the no-human-presence condition (*M* = 5.579, SD = 0.910 vs. *M* = 2.884, SD = 1.131, *t*(150) = 16.185, *p* < 0.001). This result indicates that the manipulation of human presence was successfully implemented and that the photos differed as intended in terms of human presence.

To further verify the validity of the manipulation, this study also examined participants’ perceived overall authenticity of the photos by asking whether the stadium photos realistically reflected the event scene. The results showed no significant difference in perceived overall authenticity between the human-presence condition and the no-human-presence condition (*M* = 5.579, SD = 1.203 vs. *M* = 5.621, SD = 1.243, *t*(150) = −0.199, *p* = 0.843). This finding indicates that although the photos differed in human presence, the two conditions were comparable in perceived overall authenticity, further supporting the consistency of the stimulus materials.

On this basis, this study further examined whether photo clarity and image attractiveness differed across experimental conditions. The results of the two-way ANOVA showed that, for photo clarity, the main effect of human presence was not significant (*F*(1, 148) = 0.312, *p* = 0.577), the main effect of stadium context was not significant (*F*(1, 148) = 0.426, *p* = 0.515), and the interaction between human presence and stadium context was not significant (*F*(1, 148) = 0.184, *p* = 0.668). For image attractiveness, the main effect of human presence was not significant (*F*(1, 148) = 0.287, *p* = 0.593), the main effect of stadium context was not significant (*F*(1, 148) = 0.531, *p* = 0.467), and the interaction between human presence and stadium context was not significant (*F*(1, 148) = 0.219, *p* = 0.640). These results indicate that the photos used in Study 3 did not significantly differ across conditions in non-target visual attributes, including authenticity, clarity, and image attractiveness. Therefore, the subsequent findings are unlikely to be driven by differences in photo quality or visual attractiveness.

#### 5.2.2 Main effects and interaction effects

A two-way ANOVA was conducted to examine the effects of human presence, human presence vs. no human presence, and stadium context, fan-oriented context vs. competition-oriented context, on users’ spectating intention. The results showed that human presence had a significant main effect on spectating intention (*F*(1, 148) = 35.492, *p* < 0.001). The main effect of stadium context was also significant (*F*(1, 148) = 4.480, *p* = 0.036). In addition, the interaction between human presence and stadium context was significant (*F*(1, 148) = 8.561, *p* = 0.004). These results indicate that human presence and stadium context each had independent effects on users’ spectating intention, and that the effect of human presence varied significantly across different stadium contexts.

To further examine the simple effects, *post hoc* comparisons were conducted. First, the effect of human presence on spectating intention was analyzed within each stadium context. In the fan-oriented context, stadium photos with human presence significantly increased users’ spectating intention compared with stadium photos without human presence (*M* = 5.732, SD = 0.802 vs. *M* = 4.473, SD = 1.134, *t*(74) = 5.564, *p* < 0.001). In the competition-oriented context, spectating intention was also significantly higher in the human-presence condition than in the no-human-presence condition (*M* = 5.018, SD = 0.769 vs. *M* = 4.588, SD = 0.727, *t*(74) = 2.504, *p* = 0.015). Next, the effect of stadium context was examined within each human-presence condition. Among stadium photos with human presence, users’ spectating intention was significantly higher in the fan-oriented context than in the competition-oriented context (*M* = 5.732, SD = 0.802 vs. *M* = 5.018, SD = 0.769, *t*(74) = 3.966, *p* < 0.001). However, among stadium photos without human presence, stadium context had no significant effect on spectating intention (*M* = 4.473, SD = 1.134 vs. *M* = 4.588, SD = 0.727, *t*(74) = −0.523, *p* = 0.602).

**Figure 7:**
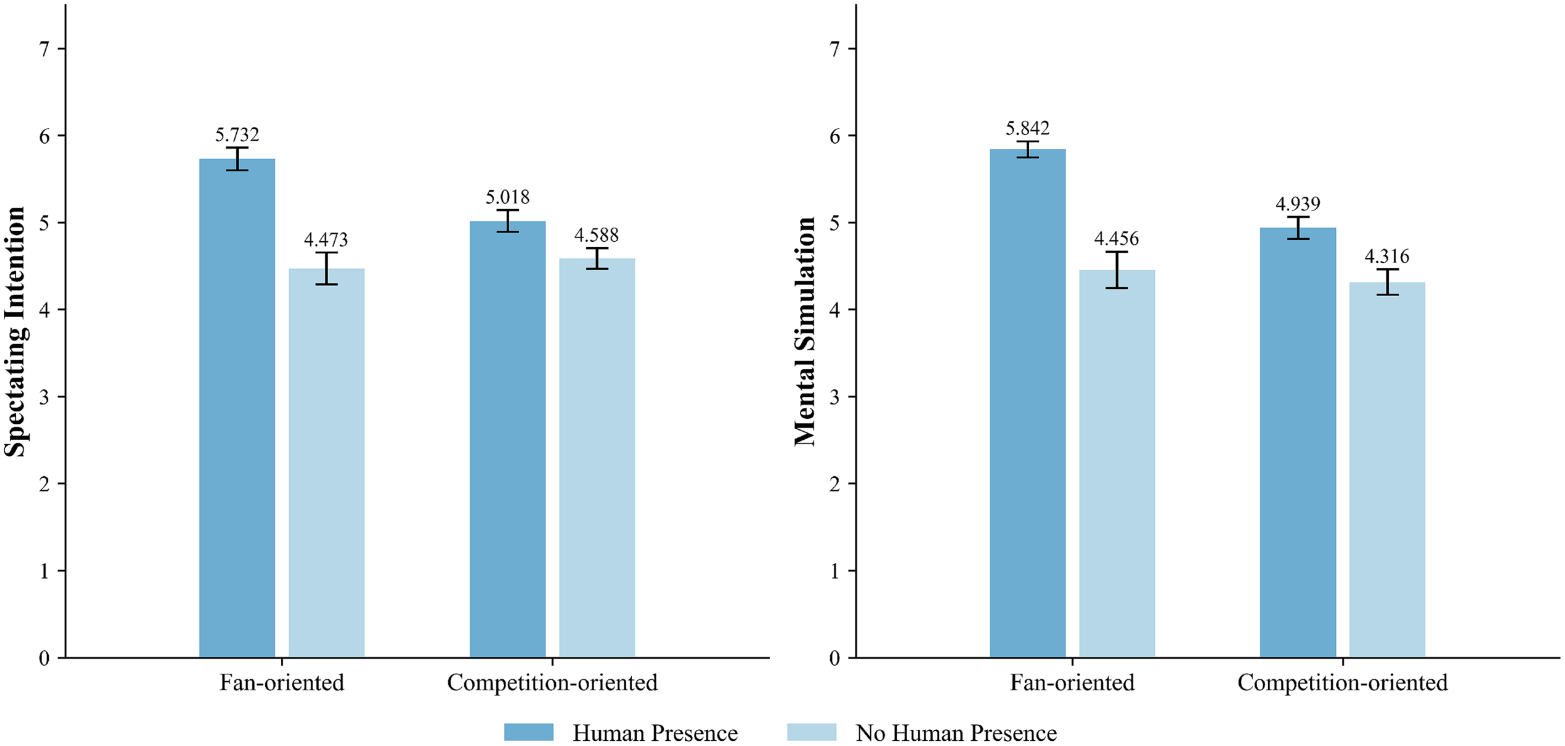
Result of Study 3.

To further examine the effects of human presence and stadium context on mental simulation, another two-way ANOVA was conducted, as shown in Figure 7. Specifically, the analysis tested the main effects of human presence, human presence vs. no human presence, and stadium context, fan-oriented context vs. competition-oriented context, as well as their interaction effect on mental simulation. The results showed that human presence had a significant main effect on mental simulation (*F*(1, 148) = 45.030, *p* < 0.001). Stadium photos with human presence elicited stronger mental simulation than photos without human presence (*M* = 5.390, SD = 0.814 vs. *M* = 4.386, SD = 1.109). The main effect of stadium context was also significant (*F*(1, 148) = 12.160, *p* < 0.001). Participants in the fan-oriented context reported higher levels of mental simulation than those in the competition-oriented context (*M* = 5.149, SD = 1.213 vs. *M* = 4.627, SD = 0.891). In addition, the interaction between human presence and stadium context was significant (*F*(1, 148) = 6.499, *p* = 0.012). These results indicate that the effect of human presence on mental simulation differed significantly across stadium contexts.

To clarify the specific effects across conditions, simple effects analyses were conducted, as shown in Figure 7. First, the effect of human presence on mental simulation was compared within each stadium context. In the fan-oriented context, stadium photos with human presence significantly enhanced participants’ mental simulation compared with photos without human presence (*M* = 5.842, SD = 0.568 vs. *M* = 4.456, SD = 1.293, *t*(74) = 6.049, *p* < 0.001). In the competition-oriented context, mental simulation was also significantly higher in the human-presence condition than in the no-human-presence condition, although the effect was weaker than that in the fan-oriented context (*M* = 4.939, SD = 0.774 vs. *M* = 4.316, SD = 0.900, *t*(74) = 3.233, *p* = 0.002). Next, the effect of stadium context was examined within each human-presence condition. Among stadium photos with human presence, mental simulation was significantly higher in the fan-oriented context than in the competition-oriented context (*M* = 5.842, SD = 0.568 vs. *M* = 4.939, SD = 0.774, *t*(74) = 5.799, *p* < 0.001). However, among stadium photos without human presence, stadium context had no significant effect on mental simulation (*M* = 4.456, SD = 1.293 vs. *M* = 4.316, SD = 0.900, *t*(74) = 0.549, *p* = 0.585).

#### 5.2.3 Moderated mediation analysis

To further clarify the mechanism through which human presence influences spectating intention, this study conducted a bias-corrected bootstrapping analysis using SPSS PROCESS Model 7 with 5,000 resamples. The analysis examined whether stadium context, fan-oriented context vs. competition-oriented context, moderated the effect of human presence, human presence vs. no human presence, on mental simulation, and further tested the mediating role of mental simulation in this process (Hayes, 2017). The direct effect of human presence on spectating intention was significant (*β* = 0.579, SE = 0.162, t = 3.573, *p* < 0.001, 95% CI [0.259, 0.899]), indicating that human presence had a significant positive effect on spectating intention.

The indirect effect through mental simulation differed across stadium contexts. Specifically, in the competition-oriented context, the indirect effect of human presence on spectating intention was significant (*β* = 0.159, BootSE = 0.086, BootLLCI = 0.025, BootULCI = 0.363). In the fan-oriented context, the indirect effect was stronger and also significant (*β* = 0.371, BootSE = 0.176, BootLLCI = 0.075, BootULCI = 0.758). The index of moderated mediation was significant (index = 0.212, BootSE = 0.126, BootLLCI = 0.023, BootULCI = 0.499), indicating a significant difference in the indirect effect between the two stadium contexts.

These results indicate that stadium context significantly moderated the process through which human presence influenced spectating intention via mental simulation. Therefore, *H3* was supported.

#### 5.2.4 Supplementary robustness analysis controlling for demographic variables

Considering that the age distribution of participants in Study 3 was relatively concentrated, this study further included age, gender, education, and income as control variables in PROCESS Model 7 and used a bootstrapping procedure with 5,000 resamples to test the moderated mediation effect. The results showed that, after controlling for these demographic variables, the conditional indirect effect in the competition-oriented context remained significant (*β* = 0.152, BootSE = 0.084, 95% CI [0.022, 0.353]). The conditional indirect effect in the fan-oriented context was also significant (*β* = 0.359, BootSE = 0.170, 95% CI [0.071, 0.733]). Furthermore, the index of moderated mediation remained significant (index = 0.207, BootSE = 0.123, 95% CI [0.021, 0.482], excluding zero). These results indicate that the moderated mediation effect in Study 3 remained robust after controlling for age, gender, educational background, and income.

## 6 Discussion

This study examined how human presence in stadium photos in social media user-generated content influences users’ spectating intention, and further tested the mediating role of mental simulation and the moderating role of stadium context. Across three experiments, the findings consistently showed that stadium photos containing people significantly increased spectating intention compared with photos without people. Mental simulation mediated this relationship, and this mediating effect was significantly stronger in fan-oriented contexts than in competition-oriented contexts. These findings suggest that the effects of sports UGC photos cannot be understood solely in terms of image quality, visual richness, or informational vividness. Instead, it is necessary to consider how human cues in images alter viewers’ self-related processing of the stadium scene. The self-referencing effect suggests that when external information is linked to individuals’ own experiences, self-concepts, or possible future behaviors, it receives deeper processing and is more likely to influence subsequent attitudes and behavioral judgments (Symons and Johnson, 1997). Mental simulation research also indicates that individuals can use external cues to mentally rehearse future situations, behavioral processes, and experiential outcomes, thereby shaping intentions and decisions (Taylor et al., 1998; Escalas, 2004; Kappes and Morewedge, 2016). In the present research, the person depicted in a stadium photo is not merely a visual object, but a social cue that helps viewers locate their own possible role as spectators. When viewers see an ordinary UGC creator, on-site spectator, or fan in the photo, they are more likely to translate the depicted position into a possible future position for themselves, thereby forming an experiential preview of watching the event on site. In Study 1, photos with human presence significantly increased spectating intention, while the stimulus control tests showed that the two photo conditions did not differ significantly in authenticity, clarity, or image attractiveness. Thus, the effect of human presence should not be attributed to the photos being more attractive, more realistic, or clearer. Rather, human cues increased the self-relevance of the stadium photos and made it easier for viewers to connect the depicted scene with their own spectating experiences and future spectator role. In this empirical framework, the self-referencing effect is not treated as a separately measured mediator, but as an upstream cognitive basis through which human presence activates mental simulation. Human cues first make the stadium photo more self-related, and mental simulation then concretizes this self-relevance into an imagined future spectating experience.

This finding should also be understood in relation to the existing literature on human presence effects. Research in digital marketing and social media imagery has not consistently shown that human presence always produces positive effects. Human cues in images may increase social media engagement, website trust, perceived service quality, or brand evaluation, but they may also generate mixed or nonsignificant effects depending on platform type, product category, human identity, and evaluation task (Hassanein and Head, 2007; Cyr et al., 2009; Naylor et al., 2012; Poor et al., 2013; Bakhshi et al., 2014; Li and Xie, 2020; Hartmann et al., 2021). Existing summaries of human presence effects suggest that their influence varies across photo type, human identity, moderators, and outcome variables (Lu et al., 2024). In experience-venue images, the presence of other people may even elicit others’ ownership, thereby reducing viewers’ preference for the venue, and this effect is shaped by experience self-identity relevance and human distinctiveness (Lu et al., 2024). The present research complements this stream of work. Sports spectating differs from experience venues such as tourist destinations, wedding venues, or restaurants, which are more likely to be understood as individualized or exclusive experiential spaces. The value of live sports events often derives from shared viewing, stadium atmosphere, emotional contagion, and audience co-presence. Therefore, people depicted in stadium photos are not necessarily interpreted as others who occupy the experience venue. They are more likely to be interpreted as people who are watching the event, or as people among whom the viewer could also become one. This difference suggests that the direction of human presence effects depends on how viewers interpret the relationship between the depicted person and the target experience. In sports UGC, the positive effect of human presence derives from role substitutability rather than from the mere presence of people. Accordingly, the incremental contribution of this research is not to show that human presence has a simple positive effect, but to demonstrate that, in sports spectating contexts, human cues are more likely to translate into spectating intention when they support self-related imagination of the spectator role.

Study 2 further revealed the psychological mechanism through which human presence affects spectating intention. The results showed that human presence significantly increased mental simulation, which in turn enhanced spectating intention. In other words, viewers did not develop stronger spectating intention simply because a person appeared in the photo. Rather, human cues helped them construct a mental scene in which they entered the stadium, watched the event, and experienced the atmosphere. Research on visual communication and consumer behavior indicates that people, actions, viewpoints, and contextual information in images can facilitate imagination of future experiences, transforming abstract consumption or participation behaviors into more concrete mental representations (Escalas, 2004; Elder and Krishna, 2012; Yim et al., 2021). In self-referencing research, future-oriented self-reference usually lacks the concrete details available in past experiences. External cues can supplement information about place, people, activities, and context, thereby facilitating the formation of consumption visions, attitudes, and behavioral intentions (Krishnamurthy and Sujan, 1999). The present research extends this logic by showing that human cues in sports UGC are not general contextual details and do not merely make the image more vivid. Rather, they provide social cues that match viewers’ future behavioral role. Empty stadiums, facilities, or isolated competition details can also provide event information, but they rarely provide a role position that viewers can enter. Photos containing ordinary spectators, UGC creators, or fan behavior make it easier for potential viewers to place themselves in a future spectating context. Thus, the contribution of this research is not to restate that additional cues facilitate mental simulation, but to identify which cues in sports UGC can transform contextual detail into self-related spectating rehearsal. Prior work has emphasized the role of contextual detail in facilitating future-oriented self-reference (Krishnamurthy and Sujan, 1999), whereas the present research further differentiates among types of contextual detail. For spectating intention, the key issue is not whether the image contains more information, but whether the image provides cues that are consistent with viewers’ future role as spectators.

Study 3 further showed that the process through which human presence influences spectating intention via mental simulation has contextual boundaries. The indirect effect was stronger in the fan-oriented context than in the competition-oriented context, but the indirect effect in the competition-oriented context still existed. This result should not be interpreted as evidence that competition-oriented contexts are ineffective. Rather, the two types of stadium context activate different contents of mental simulation. Competition-oriented contexts, or game-centered contexts, typically depict athletes, athletic actions, confrontations, and key moments. These images can help viewers imagine how the match unfolds, how athletes perform, how intense the competition is, and how exciting critical moments may be. They are therefore more likely to activate event-focused simulation, or mental simulation centered on the sporting event itself. This type of simulation helps viewers form judgments about event quality, competitive intensity, and technical appeal, but its connection to viewers’ own future spectator role is relatively indirect. Fan-oriented contexts are more likely to activate self-as-spectator simulation. People in fan-oriented contexts are usually shown entering the venue, sitting in the stands, cheering, interacting, waiting, or celebrating. These cues are more consistent with the role that potential spectators may assume in the future. Research on sports spectating indicates that spectating behavior is shaped not only by match quality and athletic content, but also by social interaction, team identification, entertainment, drama, collective emotion, and shared experience (Trail and James, 2001; Trail et al., 2003; Wann, 2006; Funk et al., 2009). Therefore, seeing athletes compete can indeed help viewers imagine how the match may unfold, but this imagination primarily concerns the event process, athletic performance, and competitive intensity. Seeing other fans watch the event, by contrast, makes it easier for viewers to imagine how they would enter the venue, watch the event, experience the stadium atmosphere, and share the event with others. The former emphasizes the visualization of event content, whereas the latter emphasizes the substitutability of the spectator role. Because the dependent variable in this research is spectating intention, and because mental simulation focuses on whether viewers can imagine themselves watching the event on site, human cues in fan-oriented contexts have stronger role congruence with the target behavior and are therefore more likely to translate mental simulation into spectating intention. Thus, the difference between competition-oriented and fan-oriented contexts is not a difference between effectiveness and ineffectiveness, but a difference in simulation object and conversion distance. Competition-oriented contexts are more closely associated with event-focused simulation, whereas fan-oriented contexts are more closely associated with self-as-spectator simulation, which has a shorter psychological distance from spectating intention.

This finding further suggests that the effect of human presence depends on the match among human role, stadium context, and target behavior. Athlete figures can enhance the visualization of event content and competitive appeal, but viewers may not necessarily imagine themselves as athletes. Fan figures are closer to the role that viewers may actually assume in the future, and therefore provide a more direct entry point for self-involvement in spectating behavior. This role-matching explanation also helps clarify why human cues may operate differently in team sports and individual sports. Team sports often have stronger shared-viewing properties, and the on-site experience is closely tied to collective cheering, group affiliation, companion interaction, and emotional resonance. Individual sports rely more strongly on individual athletic performance, technical details, key moments, speed, and competitive suspense. Accordingly, fan figures in team sports may be more likely to activate shared spectating imagination, whereas competition-oriented contexts in individual sports may be more likely to activate event-focused imagination. However, if such event-focused imagination is to be further converted into spectating intention, spectator-viewpoint cues, sideline reactions, or stadium atmosphere cues are still needed to connect athletic content with viewer experience. The interpretation of belonging should also be treated with caution. Human presence in fan-oriented contexts may contain elements of social connection, shared experience, and collective atmosphere, and research on social identity and sport fandom suggests that group identity, team identification, and shared experience influence sports spectating and fan behavior (Tajfel and Turner, 2004; Wann, 2006). However, the present research did not directly measure sense of belonging, and therefore belonging should not be treated as an empirically verified mediating mechanism. A more precise interpretation is that fan-oriented contexts provide stronger social spectating cues, making it easier for viewers to engage in self-as-spectator simulation. Future research could further examine whether loneliness and perceived similarity alter this process. Individuals with higher loneliness may be more responsive to fan interaction and on-site social atmosphere because these cues provide imagined social connection. Similarity between viewers and the people in the photo in terms of age, identity, team support, sport interest, or spectating style may also strengthen self-referential processing and role substitution (Naylor et al., 2012). In the present research, facial and identity cues were weakened in the stimuli to reduce potential confounding effects of physical attractiveness and identity recognition. Therefore, this study cannot directly test the boundary roles of similarity and loneliness. Overall, this research links human presence in sports UGC images to self-referential processing and mental simulation, showing that human cues do not simply increase image vividness. Instead, they help viewers form an experiential rehearsal related to their own future spectating behavior. Fan-oriented contexts are more likely to activate mental simulation of the self as a spectator, whereas competition-oriented contexts are more likely to activate simulation of event content. Both are theoretically meaningful, but they correspond to different contents of mental simulation.

## 7 Research implications

### 7.1 Theoretical implications

The first theoretical contribution of this study is that it clarifies the cognitive processing mechanism through which human presence in sports UGC images influences spectating intention. Existing research on sports communication and social media has paid considerable attention to the authenticity, interactivity, information diffusion, and emotional contagion of UGC content. However, less is known about how visual cues enter viewers’ self-related processing and are subsequently transformed into spectating intention. By integrating the self-referencing effect with mental simulation theory, this study shows that people depicted in stadium photos are not merely visual elements that increase image richness or vividness. Instead, they provide viewers with an assumable spectator position. The self-referencing effect suggests that when external information is connected to individuals’ own experiences, self-concepts, or possible future behaviors, it is more deeply processed and more likely to influence subsequent attitudes and behavioral judgments (Symons and Johnson, 1997). In this study, human presence transforms stadium photos from external event images into experiential objects related to viewers’ possible future spectating behavior. When viewers see ordinary UGC creators, on-site spectators, or fans in the image, they are more likely to translate the depicted position into a possible future position for themselves, thereby forming self-related imagination of watching the event. It should be emphasized that this study did not measure the self-referencing effect as a separate mediator. Rather, it conceptualized self-referencing as the upstream cognitive basis through which human presence activates mental simulation. Human cues first increase the self-relevance of stadium photos, and mental simulation then concretizes this self-relevance into an imagined psychological scene of watching the event on site. This theoretical specification explains how human presence enters viewers’ psychological processing and avoids treating self-referencing and mental simulation as two simply parallel mechanisms.

The second theoretical contribution of this study is that it advances mental simulation theory from a general visual vividness explanation to a role-matching explanation. Existing research on mental simulation indicates that images, perspectives, actions, and contextual cues can help individuals mentally rehearse future behaviors or consumption experiences, thereby shaping attitudes and behavioral intentions (Escalas, 2004; Elder and Krishna, 2012; Yim et al., 2021). In anticipatory self-referencing, future experiences often lack sufficient contextual detail. Contextual detail in advertising can help consumers form more complete consumption visions and enhance attitudes and behavioral intentions (Krishnamurthy and Sujan, 1999). Building on this logic, the present study provides a more specific theoretical refinement. Human cues in sports UGC are not merely additional contextual details, nor are they simply visual elements that increase the amount of information. They are social cues that match viewers’ future behavioral role. Empty venues, facilities, partial stadium scenes, or competition backgrounds can also provide contextual information, but they rarely provide a position that viewers can easily assume. By contrast, photos containing ordinary spectators, UGC creators, or fan behaviors make it easier for potential viewers to place themselves into a future spectating scene. Thus, the contribution of this study is not to show again that more contextual detail facilitates mental simulation. Rather, it identifies which contextual cues in sports UGC are more likely to be transformed into self-related spectating rehearsal. For spectating intention, the critical issue is not whether the image contains more information, but whether it provides assumable cues that are congruent with viewers’ future spectator role.

The third theoretical contribution of this study is that it situates sports UGC research within the literature on human presence effects in digital marketing and further clarifies the contextual dependency of human presence effects. Research on digital marketing and social media imagery has shown that human presence is not a uniformly positive variable. Human cues may increase social media engagement, website trust, social presence, or brand evaluation, but they may also produce mixed or nonsignificant effects depending on product type, platform environment, human identity, and evaluation task (Bakhshi et al., 2014; Cyr et al., 2009; Hartmann et al., 2021; Hassanein and Head, 2007; Li and Xie, 2020; Naylor et al., 2012; Poor et al., 2013). In identity-relevant experience venues, other people depicted in a photo may elicit others’ ownership and thereby reduce viewers’ liking and preference for the venue (Lu et al., 2024). This finding indicates that people in images do not inherently possess persuasive advantages. Their effects depend on how viewers interpret the relationship between the depicted person and the target experience. The present study complements this literature. Sports spectating differs from tourist destinations, wedding venues, restaurants, and other experience venues that may be interpreted in a more individualized or exclusive manner. The value of live sports events often derives from shared viewing, stadium atmosphere, collective emotion, and audience co-presence. Therefore, people in stadium photos are not necessarily interpreted as others occupying the experience venue. They are more likely to be interpreted as people watching the event or as people among whom the viewer could also become one. Accordingly, this study does not simply show that human presence has a positive effect in sports UGC. It further demonstrates that human presence is more likely to be translated into spectating intention through self-referential mental simulation when human cues match viewers’ future behavioral role. This finding provides contextual evidence from sports consumption for explaining why human presence effects are inconsistent in existing digital marketing research.

The fourth theoretical contribution of this study is that it distinguishes the different contents of mental simulation activated by game-centered contexts and fan-oriented contexts, thereby providing a more refined explanation of the moderating mechanism in Study 3. Study 3 showed that the indirect effect of human presence on spectating intention through mental simulation was stronger in the fan-oriented context than in the competition-oriented context, although the indirect effect in the competition-oriented context remained significant. This result should not be understood as indicating that competition-oriented contexts are ineffective. Rather, the two types of stadium context activate different objects of mental simulation. Competition-oriented contexts typically depict athletes, athletic actions, confrontations, and key moments. They help viewers imagine how the match unfolds, how athletes perform, how intense the event is, and how exciting key moments may be. This type of mental simulation is closer to event-focused simulation, namely mental simulation centered on event content. It can strengthen viewers’ judgments of event quality, competitive intensity, and technical appeal, but its connection with viewers’ own future spectator role is relatively indirect. Fan-oriented contexts are more likely to activate self-as-spectator simulation. People in fan-oriented contexts are usually shown entering the venue, sitting in the stands, cheering, interacting, waiting, or celebrating. These cues are more consistent with the role that potential viewers may assume in the future. When viewers see other fans watching the event, they are more likely to translate the depicted human position into their own future position and imagine themselves sitting in the stands, watching the event with others, and experiencing the stadium atmosphere. Research on sports spectating motivations also suggests that spectating behavior is driven not only by match quality and athletic content but also by social interaction, team identification, entertainment, collective emotion, and shared experience (Funk et al., 2009; Trail and James, 2001; Trail et al., 2003; Wann, 2006). Therefore, athlete figures can help viewers form event-focused simulation, whereas fan figures are more likely to help viewers form self-as-spectator simulation. Both types of human presence can elicit mental simulation, but the simulated objects differ. Because this study focuses on spectating intention, the type of simulation that is more congruent with the spectator role is more likely to generate behavioral conversion. This distinction provides a more nuanced theoretical account of how stadium context shapes the psychological effects of sports UGC images.

Finally, this study extends the theoretical boundary of research on visual communication in sports UGC. Prior research on sports-related social media content has mainly focused on user interaction, content engagement, fan identity, and platform-level communication effects. Less attention has been paid to how the relationship among image cues, viewer roles, and future experience rehearsal explains the formation of spectating intention. The present study shows that sports UGC is not merely a visual material for transmitting event information or recording on-site atmosphere. It can also function as a psychological trigger that guides viewers to locate themselves in the scene and imagine future spectating experiences. The effect of human presence depends on the match among human role, stadium context, and target behavior. Fan figures are more likely to provide an entry point into the spectator role, whereas competition figures are more likely to provide an entry point into event content. Both are theoretically meaningful, but they correspond to different contents of mental simulation. This study therefore shifts sports UGC research from the question of whether visual content is effective to the question of which human cues influence spectating intention through which forms of mental simulation. It also provides a theoretical basis for future research on boundary conditions such as human similarity, loneliness, fan identity, sport type, and platform content format.

### 7.2 Practical implications

The empirical findings of this study provide more targeted practical guidance for the operation of sports UGC in social media environments. Study 1 showed that stadium photos containing people generated stronger spectating intention than stadium photos without people. Study 2 further demonstrated that this effect operated through mental simulation. Study 3 showed that the effect of human presence on spectating intention through mental simulation was stronger in fan-oriented contexts, whereas the effect in competition-oriented contexts was relatively weaker but still present. Accordingly, the practical value of sports UGC should not be reduced to the simple recommendation that images with people should be used more often. A more precise strategy is to evaluate whether the people depicted in an image provide users with an assumable spectator position and whether they help users imagine how they might enter the venue, watch the event, and experience the stadium atmosphere in the future. Existing research also suggests that the effect of human presence in online images is not always uniformly positive, but varies depending on photo type, human identity, experiential attributes, and viewers’ psychological needs. Therefore, when selecting sports UGC, event organizers, club media teams, and platform operators should not rely only on whether an image contains people, whether it is visually appealing, or whether it has already received high engagement. Instead, experiential simulability should be treated as an important criterion for content selection.

In content curation and event communication, UGC containing people should primarily serve the construction of spectating experience. The findings indicate that the effect of human presence mainly derives from its activation of mental simulation. This means that high-value UGC is not simply a photo that displays stadium space, event facilities, or athletic movements, but a visual material that helps potential viewers form first-person imagination of spectating. Future-oriented self-referencing often requires concrete contextual cues. People, places, activities, and interactive details in a scene help individuals construct more complete mental representations of future experiences. Accordingly, when reposting UGC, producing event preview posts, selecting short-video covers, or designing social media posters, event organizers should prioritize images that present spectators’ postures, on-site interactions, emotional expressions, and viewing positions. For example, images showing audience entry, waiting in the stands, collective cheering, post-game celebration, and interaction with companions can make it easier for users to place themselves into the stadium scene. By contrast, empty-venue photos, facility photos, or partial match images lacking human participation cues can still convey event information, but their capacity to stimulate spectating intention is relatively limited. Such images are more suitable for venue introduction, schedule communication, or event recap.

For fan-oriented contexts, the practical focus should be placed on spectating conversion. Study 3 showed that the indirect effect of human presence on spectating intention through mental simulation was stronger in fan-oriented contexts. This suggests that when images show other fans watching, cheering, interacting, or celebrating, potential viewers are more likely to imagine themselves as on-site spectators and to form an expectation that they could also participate in the experience. Research on sports spectating motivation also indicates that event viewing is not driven only by match quality and athletic performance. Social interaction, excitement, collective emotion, and shared experience also shape spectators’ event participation behavior. Therefore, in ticket promotion, live-stream reservations, event previews, and urban sports-event campaigns, event organizers should make greater use of fan-oriented UGC containing people. Such content can transform the event from an object to be watched into a social situation that can be entered, shared, and experienced, making it more suitable for promoting spectating intention.

For competition-oriented contexts, the practical value should not be understood as low. Although the indirect effect in competition-oriented contexts was weaker than that in fan-oriented contexts in Study 3, it remained present. This indicates that game-centered contexts can still influence spectating intention through mental simulation, but the simulated content is more oriented toward the event process than toward the viewer’s own spectator role. Athlete movements, confrontation moments, technical performance, and key plays in competition-oriented images can help users imagine how the match unfolds, whether the event is intense, and how impressive the athletes’ performance may be. Such content is more suitable for displaying event professionalism, maintaining core fans, communicating match updates, highlighting key moments, and popularizing sport-specific knowledge. For highly involved fans or users who primarily focus on athletic content, images of people in competition-oriented contexts remain valuable. However, when the communication goal is to attract potential viewers to attend in person or watch a live broadcast, it is insufficient to display only athletes and athletic actions. A more effective approach is to incorporate spectator viewpoints, on-site reactions, or stand atmosphere into competition-oriented content, so that users can perceive the athletic content while also imagining themselves as spectators watching that moment.

Based on the findings of Study 3 regarding fan-oriented and competition-oriented contexts, this strategy may apply differently across different types of sports. For team sports, live spectating is often accompanied by stronger shared emotions, group affiliation, and fan interaction. Therefore, human presence in fan-oriented UGC may be more effective in activating shared spectating imagination. For conversion-oriented communication in sports such as football, basketball, volleyball, and ice hockey, more content can present stand interaction, collective cheering, pre-game gathering, and post-game celebration, enabling potential viewers to perceive that they can enter a shared viewing and emotional experience. Athlete images in competition-oriented contexts remain suitable for match updates and highlight communication, but if the goal is to stimulate spectating intention, they should be combined with fan reactions and stadium atmosphere. For individual sports, spectating appeal often comes more from athletes’ performance, technical actions, and key moments. Thus, competition-oriented contexts themselves retain high communication value. Sports such as tennis, track and field, swimming, combat sports, skiing, and racing can present their viewing appeal through athletic performance, key rounds, speed, and competitive suspense. However, if the goal is to promote spectating intention, athletic content still needs to be connected with spectator-stand viewpoints, sideline reactions, or stadium atmosphere cues, so that users not only imagine how the event unfolds but also imagine how they themselves would watch the event.

In social media platforms and event account operations, this study further suggests that content distribution logic should shift from exposure-oriented ranking to psychological conversion-oriented ranking. Likes, comments, and shares can reflect content popularity, but highly engaged content does not necessarily possess high potential for spectating conversion. When identifying high-value sports UGC, platforms and event accounts can attend to image features that are closer to mental simulation, such as human visibility, spectator viewpoint, emotional expression, interaction intensity, and on-site atmosphere. For potential viewers or low-involvement users, fan-oriented UGC containing people can be prioritized to reduce psychological distance between users and the event. For core fans or highly involved users, more content featuring athlete performance and key moments in competition-oriented contexts can be provided to satisfy their interest in competitive quality and event information. Such content configuration allows different types of UGC to serve different communication goals. Fan-oriented contexts primarily support spectating conversion, whereas competition-oriented contexts primarily support event-content display and core-fan maintenance. Event organizers can also use topic design to guide fans to produce UGC with stronger experiential simulability, such as encouraging users to share first-person spectating photos, entry moments, stand interactions, reactions to key moments, and post-game emotions. In this way, sports UGC is no longer merely supplementary material for event communication, but can become an important resource linking social media browsing, mental simulation, and spectating intention.

## 8 Limitations and future research

This study focused on human presence in sports UGC stadium photos, mental simulation, and spectating intention. Across three experiments, the findings provide evidence for understanding how visual content influences sports spectating intention. Although the results were consistent across studies, and supplementary analyses further controlled for demographic variables, several limitations remain and should be examined in future research.

First, the sample was mainly drawn from Chinese social media users, and participants aged 31 to 40 accounted for a relatively high proportion across the three studies. Although this age group has relevant sports consumption experience and social media use experience, and thus provides a valid sample for the research questions, the relatively concentrated age distribution may limit the generalizability of the findings to other age groups. Different age groups may differ in sports preferences, social media use patterns, understanding of UGC content, perceived cost of offline spectating, and sensitivity to human cues. Younger users may be more familiar with immersive forms of expression on short-video platforms and image-based social media, whereas older users may rely more on event information, acquaintance recommendations, or traditional media content. Future research could use a more balanced age structure and further compare user responses across different cultural and platform environments. Fan culture, collective spectating habits, social media interaction norms, and sports consumption patterns in different countries and regions may influence the strength of the effects of human presence on mental simulation and spectating intention. Cross-cultural samples and cross-platform data would further help test the external validity of the present findings.

Second, this study mainly examined two types of visual factors, namely human presence and stadium context. This focus helped identify core image cues in sports UGC, but it also simplified the complex visual information contained in stadium photos. In real social media environments, stadium UGC often includes multiple cues at the same time, such as camera angle, shooting distance, number of people, bodily posture, facial expression, spectator density, spatial layout of the venue, color saturation, and perceived motion. These factors may jointly influence viewers’ immersion, emotional responses, and mental simulation intensity. Existing research on visual marketing and mental simulation has shown that visual perspective, human action, and contextual details can influence individuals’ imagination of future experiences and behavioral judgments (Bone and Ellen, 1992; Schlosser, 2003; Libby et al., 2007). In the present study, the stimuli were standardized, and non-target visual attributes were tested to control for perceived authenticity, clarity, and image attractiveness as much as possible. Future research could further adopt more fine-grained visual variable designs or use computer vision methods to quantify features such as human area, visual salience, facial visibility, action intensity, and emotional intensity. Such work would help clarify which specific visual attributes in sports UGC are most likely to facilitate self-related spectating imagination.

Third, the dependent variable in this study was spectating intention rather than actual spectating behavior. Behavioral intention reflects individuals’ psychological tendency under a specific stimulus and is appropriate for testing the causal relationship among human presence, mental simulation, and spectating intention. However, actual spectating behavior may also be affected by multiple situational factors, such as ticket price, time arrangement, venue distance, companion participation, event accessibility, weather conditions, and live-streaming alternatives. Although behavioral intention is theoretically related to actual behavior, the two are not equivalent. Actual behavior is often shaped by perceived behavioral control, situational constraints, and external opportunities (Sheeran, 2002; Webb and Sheeran, 2006). Future research could combine platform logs, click behavior, live-stream reservations, ticket purchase records, coupon redemption, event-page dwell time, or actual attendance data to examine whether sports UGC photos can extend from spectating intention to real behavioral conversion. Social media A/B testing or field experiments could also be used to compare the effects of different types of stadium photos on click-through rates, reservation rates, ticket purchase rates, and content-sharing behavior. Such research would further improve the ecological validity and behavioral predictive value of the findings.

Fourth, this study only distinguished between fan-oriented contexts and competition-oriented contexts. Although this distinction helped test the basic contextual boundary of human presence effects, the context of sports events is far more complex. Event scale, competition level, sport type, team popularity, fan base, and historical significance of the match may all change how users process stadium photos. In particular, team sports and individual sports may involve different spectating experience structures. Team sports usually place greater emphasis on shared viewing, group affiliation, collective emotion, and fan interaction, whereas individual sports rely more on athlete performance, technical details, key moments, and competitive suspense. Research on sports spectating motivation also indicates that spectator consumption is not driven solely by the event itself, but is also related to social interaction, team identification, entertainment experience, and emotional experience (James and Ross, 2004; Robinson et al., 2005; Yoshida et al., 2013). Therefore, the effects of human presence and stadium context may vary across sport types. Future research could compare team sports, such as football, basketball, and volleyball, with individual sports, such as tennis, track and field, swimming, and combat sports, to examine the relative roles of fan figures, athlete figures, and venue environment cues across different sport contexts. Such extensions would further clarify the applicability of the present findings across different sports.

Finally, although the theoretical explanation in this study suggests that fan-oriented contexts may involve shared experience, social connection, and collective atmosphere, the present research did not directly measure sense of belonging. Therefore, belonging should not be treated as an empirically verified mediating mechanism. A more cautious interpretation is that fan-oriented contexts provide stronger social spectating cues, making it easier for viewers to engage in self-as-spectator simulation. Future research could further examine the boundary roles of loneliness and perceived similarity. Individuals with higher loneliness may be more responsive to fan interaction, collective cheering, and on-site social atmosphere, because these cues may provide imagined social connection and shared experience (Baumeister and Leary, 2017; Smith et al., 2021). Similarity between viewers and the people in the photo in terms of age, gender, identity, supported team, sport interest, or spectating style may also enhance self-referential processing and role substitution. Self-congruity and similarity cues may influence individuals’ evaluations and behavioral responses, suggesting that similarity between the depicted person and the viewer may also shape the human presence effect in sports UGC (Aguirre-Rodriguez et al., 2012). Conversely, when the person in the photo is perceived as dissimilar to the viewer, or when the person’s identity is too distinctive, the human cue may be interpreted more as another person’s experience rather than as a future self-relevant scenario. Future research could systematically manipulate loneliness and perceived similarity while controlling for physical attractiveness and photo quality, thereby further explaining why different users may generate different levels of mental simulation and spectating intention in response to the same human cue.

## Data Availability

The raw data supporting the conclusions of this article will be made available by the authors, without undue reservation.
